# Emotional Vulnerability in Adolescents (EVA) Longitudinal Study: Identifying individual differences in symptoms of adolescent depression and anxiety and their biopsychosocial mechanisms based on demographic and mental health characteristics

**DOI:** 10.12688/wellcomeopenres.22685.2

**Published:** 2025-09-01

**Authors:** Asnea Tariq, Elaine Gray, Alice M. Gregory, Stella W. Y. Chan

**Affiliations:** 1 University of Reading School of Psychology and Clinical Language Sciences, Reading, England, RG6 6ES, UK; 2 The University of Edinburgh School of Health in Social Science, Edinburgh, Scotland, EH8 9AG, UK; 3 Department of Psychology, Goldsmiths University of London, London, England, SE14 6NW, UK

**Keywords:** Depression, Well-being, Adolescent Mental health, Biopsychosocial risk factors, Risk and Resilience

## Abstract

**Background:**

Adolescent depression and anxiety are highly prevalent, recurrent, and disabling mental health conditions. Current treatment outcomes are suboptimal, often leaving young people with residual symptoms and high relapse rates. To inform future development of more effective preventative strategies, the Emotional Vulnerability in Adolescents (EVA) study aimed to identify vulnerability markers for adolescent depression and anxiety. Specifically, it examined the associations between mental health outcomes and potentially modifiable biopsychosocial factors. The present report provides an overview of the study design and methodology, summarised the demographic, clinical, and mechanistic characteristics of the sample, and examined individual differences by age, gender, and personal and familial history of mental health at baseline.

**Methods:**

Data collection was conducted across three-time points (baseline, 6-months and a 60-month follow-up). A total of 425 adolescents (60.5% female) aged 12 -18 years (Mean = 15.06, SD = 1.75) were recruited at baseline. A comprehensive battery of measures to assess a range of bio-psycho-social factors was employed.

**Results:**

We replicated previous findings in suggesting that females and those with a personal or familial history of mental health difficulties have higher levels of depression and anxiety and lower levels of well-being. These vulnerable sub-groups were also found to differ from their counterparts in a number of biopsychosocial factors; specifically they showed poorer sleep quality, lower levels of resilience, and higher levels of rumination, stress, neuroticism, external shame, bullying experiences, neural-cognitive biases, and dysfunctional attitudes. Furthermore, symptoms of depression and anxiety increased with age and peaked around age 15; age was also associated with an increased risk for eating disorders.

**Conclusions:**

The present findings highlight the importance of considering individual differences in developing future preventative and intervention strategies by targeting underlying mechanisms that are more specifically prominent in each individual subgroup of the population.

## Plain language summary

Depression and anxiety are common and disabling mental health conditions in adolescents that are likely to persist for long periods if left untreated. Current treatments are not always effective, leaving many young people with ongoing symptoms and at risk of relapse. To help create better prevention strategies, the Emotional Vulnerability in Adolescents (EVA) study examined various factors that could make adolescents more vulnerable to depression and anxiety. This report provides an overview of the study’s design, the characteristics of the participants, and differences based on age, gender, and mental health history. Data was collected at three different times: the start of the study (baseline), after 6 months, and after 60 months. A total of 425 adolescents, mostly females, aged 12 to 18 years participated. The participants completed various assessments covering biological, psychological, and social factors. The study confirmed that females and those with personal or family histories of mental health issues had higher levels of depression and anxiety and lower levels of well-being. These groups also had poorer sleep, less resilience, and more rumination, stress, neuroticism, external shame, bullying experiences, cognitive biases, and dysfunctional attitudes. Symptoms of depression and anxiety increased with age, peaking around age 15, and age was also linked to a higher risk of eating disorders. The findings suggest that future prevention and intervention strategies should consider individual differences, focusing on specific underlying factors that are more prominent in each subgroup.

## 1. Introduction

### 1.1. Adolescence: A period of increased vulnerability for mental ill health

Adolescence is a unique and formative developmental phase marked by physical, social, biological, and emotional changes ( WHO, 2021). Although many young people transition into healthy adulthood, this developmental stage is also associated with an increased vulnerability to mental health problems ( Blakemore, 2019). Globally, it is estimated that nearly 14% of adolescents (1 in 7) are likely to experience mental health difficulties that often remain unrecognised and untreated ( WHO, 2021). Furthermore, research suggests that approximately 50% of adolescents will have experienced at least one episode of mental health problem by the age of 14, with 75% of young people experiencing mental health difficulties by the age of 24 ( Jurewicz, 2015; Kessler *et al.*, 2007; Kim-Cohen *et al.*, 2003; McGorry & Mei, 2018; NICE guidelines, 2015). In the UK, mental health problems affect nearly 7.5 million adolescents, corresponding to 12.8 % of the UK ( UNICEF, 2021).

### 1.2. Depression and anxiety among adolescents

Depression and Anxiety are two distinct yet highly comorbid mental health conditions with overlapping symptoms ( Goodwin, 2015). Recent reports from UNICEF’s State of World’s Children highlight that 13% of adolescents aged of 10–19 globally live with an undiagnosed mental health issue, with depression and anxiety constituting 40% of all mental health disorders ( UNICEF, 2021). Statistical estimates further suggest that 1 in 5 individuals will experience an anxiety and/ or depressive disorder by the age of 25, contributing to 45% of the global burden of diseases ( Colizzi *et al.*, 2020; Mokdad *et al.*, 2016; Santomauro *et al.*, 2021). These disorders are not only distressing and impairing but also prevalent among adolescents ( Garber & Weersing, 2010; WHO, 2017; Yu *et al.*, 2022). Mental health problems in adolescents can interfere with daily functioning, interpersonal relationships, academic performance, employment opportunities, and increase healthcare costs ( WHO, 2021). They also predict more severe and complex symptomology, recurrent mental health episodes, increased suicide risks, and co-occurring psychiatric or physical disorders across the life span ( Garber & Weersing, 2010; WHO, 2021).

### 1.3. Treatments for adolescent depression and anxiety

Encouragingly, findings from clinical trials indicated that some adolescents with depression and anxiety achieve partial or full remission through combined, optimised therapies such as Cognitive Behavioural Therapy (CBT), behavioural activation, problem-solving, interpersonal, third-wave psychotherapies, and/ or antidepressant medications ( Cox *et al.*, 2012; Cuijpers *et al.*, 2023; Robberegt *et al.*, 2022). A recent meta-analysis of 38 psychotherapeutic treatments reported a 50% reduction in depressive symptoms in children and adolescents, with a 39% response rate within two (±1) months of baseline ( Cuijpers *et al.*, 2023). Similarly, pooled data from 22 studies suggested a significant post-treatment improvement in functioning among anxious adolescents, with a large effect size (Cohen's d = 1.55).

Despite these improvements, treatment effectiveness in younger individuals lags behind that in adults. Cuijpers and colleagues (2023) reported a significantly smaller effect of psychotherapy for children (Hedges *'g'* = 0.35) and adolescents (Hedges *'g'* = 0.55) compared to adults (Hedges *'g'* = 0.66 - 0.98). The burden of these disorders remain high due to reduced quality of life ( Lepine & Briley, 2011) and a substantial risk of relapse (i.e., 39–72%; Bockting *et al.*, 2015; Cox *et al.*, 2012; Vos *et al.*, 2020). Even after remission, around 50% of young people continue to experience disabling residual symptoms ( Cox *et al.*, 2012; Kennard *et al.*, 2006). The relapse rates for depressive disorders among adolescents range from 47% to 67% over 6 to 24 months ( Bockting *et al.*, 2015; Lepine & Briley, 2011), with a 72% chance of recurrence over a 15-year period ( Bockting *et al.*, 2015). Alarmingly, the risk of developing a chronic depressive illness increases by about 60% after experiencing two or more major depressive episodes ( Bockting *et al.*, 2015). For anxiety disorders, the likelihood of relapse or recurrence varies between 39% and 58% over a 12-year period ( Batelaan *et al.*, 2017; Scholten *et al.*, 2016). Another study estimated a 48% relapse risk among adolescents within four years ( Ginsburg *et al.*, 2014), often with the crossover into major depression or other anxiety disorders later in life ( Robberegt *et al.*, 2022; Scholten *et al.*, 2016).

### 1.4. The imperative of early intervention

Given the high prevalence and persistence of depression and anxiety in adolescence, evidence strongly suggests that untreated and unresolved mental health problems in this period often extend into young adulthood and beyond ( Colizzi *et al.*, 2020; McGorry & Mei, 2018). Simultaneously, adolescence has been recognised as a sensitive neurodevelopmental window with the potential to promote lifelong well-being and psychological resilience ( Marco *et al.*, 2011). Thus, implementing early feasible, efficient, and cost-effective intervention strategies is crucial to reduce long-term and recurrent effects of these disorders ( Colizzi *et al.*, 2020; McGorry & Purcell, 2009).

Improved detection of early symptoms, timely intervention, effective treatment, and prevention of relapse or recurrence require the identification of reliable vulnerability markers. These markers might help predict illness onset and track the developmental trajectories of risk and resilience ( Department of Health UK, 2017). Addressing these challenges is not only of scientific and clinical importance, but also a key priority for public health and societal well-being ( Department of Health UK, 2017).

### 1.5. Interplay of multifactorial risk and protective factors for adolescents mental health

A substantial body of literature supports a multifactorial causal model for adolescents’ mental health, encompassing biological, health and lifestyle, psychological, cognitive, and interpersonal/social risk factors ( Kieling *et al.*, 2011; Patel *et al.*, 2007).

***1.5.1. Interpersonal and social risk factors*.** During adolescence, young people develop key social and emotional skills essential for lifelong health and well-being, such as adopting a healthy lifestyle, engaging in physical activity, learning effective coping strategies, and developing interpersonal and emotional regulation skills ( Colizzi *et al.*, 2020; McGorry & Mei, 2018; WHO, 2021). In contrast, social and environmental stressors such as adversity, poverty, income inequality, identity conflicts, peer pressure, poor family environment and parenting practices, conflictual peer relationships, violence, and abuse have been consistently highlighted as major risk factors for adolescent mental health problems ( Patel *et al.*, 2007; WHO, 2021). Additionally, adolescents with parental histories of mental health disorder or substance misuse are at increased risk of developing mental illnesses themselves ( Patrick *et al.*, 2020).

***1.5.2. Health and lifestyle risk factors*.** In addition to the above, various health and lifestyle risk factors, including *Body Mass Index* (BMI; Gallagher *et al.*, 2023; Hoare *et al.*, 2016; WHO, 2023), *eating attitudes* ( Gallagher *et al.*, 2023; WHO, 2023), and *social media usage* ( Alonzo *et al.*, 2021; O'Relly *et al.*, 2018) have been identified as significant precursors of adolescent depression and anxiety. BMI is defined as a weight-for-height measure, with adolescents in the top or bottom 5% of age- and sex-adjusted percentiles classified as overweight or underweight, respectively ( Hales *et al.*, 2018). Global data indicate that 1 in 6 adolescents are now classified as obese, with obesity increasing from 4% to 18% over the past three decades ( Nicolucci & Maffeis, 2022; WHO, 2023). Longitudinal studies have shown a bidirectional relationship between obesity and increased depression and anxiety symptoms among adolescents ( Hoare *et al.*, 2016; Luppino *et al.*, 2010). These findings have also highlighted several other relevant factors, such as poor physical activity, unhealthy diet, disordered eating attitudes, and sleep disturbances, which may potentially mediate these significant associations ( Lindberg *et al.*, 2020).

Disordered eating attitudes, such as maladaptive beliefs, thoughts, feelings, and behaviours related to food, are also established risk factors for adolescents' depression and anxiety ( Costarelli *et al.*, 2011; Hayes *et al.*, 2018). Comorbidity is common, with eating disorders and mood disorders frequently co-occurring and each influencing the onset and course of the course ( Calvo-Rivera *et al.*, 2022; Hambleton *et al.*, 2022; McGrath *et al.*, 2020). These findings highlight the importance of including assessments of eating attitudes when evaluating mood symptoms in adolescence.

The rise of social media presents another emerging concern ( Kelly *et al.*, 2019; O'Reilly, 2020). A recent survey highlighted that nearly all adolescents use online platforms, with 97% of US teens (ages 13–17) reporting use of at least one major platform and 99% of UK teens using social media for at least 21 hours per week ( Ofcom, 2023). High social media usage, particularly among adolescent girls, has been associated with increased symptoms of depression and anxiety Chochol *et al.*, 2023; Kelly *et al.*, 2019). It has been suggested that constant exposure to social media information, carefully curated images, social comparisons, and pressure to conform to unrealistic standards may contribute to feelings of inadequacy, low self-esteem, a fear of missing out, and emotional distress ( Kelly *et al.*, 2019; Vidal *et al.*, 2020). Nevertheless, given the complex and evolving nature of this literature, caution is warranted in making broad generalisations.

***1.5.3. Personality and Coping Factors*.** In addition to the health and lifestyle factors, personality traits, and coping factors, such as *Neuroticism* ( Chan *et al.*, 2007; Liu *et al.*, 2020), *Resilience* ( Anderson & Priebe, 2021), and *Stress* ( Thapar *et al.*, 2012), are strongly associated with mental health outcomes in adolescents. Neuroticism – characterised by a heightened tendency to experience negative emotions and intense stress response – has been consistently linked to adolescent depression and anxiety ( He *et al.*, 2021; Lahey, 2009; Liu *et al.*, 2020; Navrady *et al.*, 2018). Individuals high in neuroticism often engage in maladaptive coping strategies, such as avoidance or rumination, contributing to elevated psychological distress ( Cho *et al.*, 2017; Ueda *et al.*, 2018).

Resilience, conceptualised as the capacity to maintain or regain mental health in the face of adversity, has gained attention as a protective factor ( Gloria & Steinhardt, 2016; Kieling *et al.*, 2011). Recent empirical findings suggest that resilience may buffer the effects of neuroticism and stress, and in inversely associated with depression risk ( Navrady *et al.*, 2018). Strengthening resilience and promoting positive emotions may enhance recovery and long-term outcomes, suggesting a need to shift some intervention efforts beyond symptom alleviation towards broader well-being enhancement ( Navrady *et al.*, 2018).

***1.5.4. Psychosocial Risk Factors*.** From a psychosocial perspective, negative social experiences such as bullying, rejection, social isolation, withdrawal, shame, and guilt are significantly associated with adolescent depression and anxiety ( Parker & Roy, 2001; Rosenblat *et al.*, 2019). Interestingly, the relationship appears to be bidirectional, with depressed adolescents often perceiving their environment as more hostile and unsupportive ( Parker & Roy, 2001). Additionally, poor emotional regulation has been linked with both anxiety ( Cisler *et al.*, 2010; Schneider *et al.*, 2018) and depression ( Poon *et al.*, 2016). Conversely, the presence of adequate social support has been found to protect against poor psychological outcomes.

***1.5.5. Cognitive risk factors*.** Cognitive style is another critical domain influencing adolescent mental health. Negative cognitive biases – including those in attention, memory, and interpretation – have been identified as risk factors for the onset and maintenance of depression and anxiety symptoms ( Craske & Pontillo, 2001; Gotlib & Joormann, 2010; Smith *et al.*, 2018). Key maladaptive traits include low self-esteem ( Sowislo & Orth, 2013), high self-criticism ( McIntyre *et al.*, 2018), excessive rumination ( Michl *et al.*, 2013), attributional biases ( Smith *et al.*, 2018), and dysfunctional attitudes ( Yapan *et al.*, 2022). These biases contribute to difficulties in processing and disengaging from negative stimuli, and they often persist across various conditions ( Gotlib & Joormann, 2010).

Cognitive vulnerabilities are increasingly viewed as transdiagnostic factors rather than symptoms specific to one disorder ( Yapan *et al.*, 2022). While attention and interpretation biases have been strongly associated with adolescent depression ( Smith *et al.*, 2018), memory bias appears less influential in the youth population than in adults ( Platt *et al.*, 2017). Besides, some findings suggest that interpretation bias may be more closely linked with anxiety, whereas memory bias may relate more to depressive symptoms, though further adolescent-specific research is needed ( Leung *et al.*, 2022; Smith *et al.*, 2018).

***1.5.6. Biological risk factors*.** Biological marker, such as cortisol levels, have been recognised as the ‘stress hormone’ and a potential predictor of adolescent depression and anxiety ( Guerry & Hastings, 2011). There are different ways to measure cortisol, with recent research suggesting that hair cortisol can be a reliable alternative measurement to conventional salivary measures (assessed via saliva or blood) due to its lower collection burden ( Short *et al.*, 2016). However, findings regarding the relationship between cortisol levels and depressive and anxiety symptoms have been mixed. While some studies found no linear association between hair cortisol and depressive symptoms ( Ford *et al.*, 2019; Kische *et al.*, 2021), others reported a curvilinear association, where both low and high cortisol levels were associated with increased depressive symptoms ( Ford *et al.*, 2019). For anxiety symptoms, no significant associations with cortisol concentrations have been consistently observed ( Xu *et al.*, 2019).

### 1.6. Rationale of the present report

Taken together, the current available evidence has strongly argued for the role of multiple psychological, social, cognitive, personality, lifestyle, and biological factors as vulnerability markers for adolescents' depression and anxiety. However, sparsely available research has empirically evaluated these domains in a homogeneous adolescent sample. The present study – Emotional Vulnerability in Adolescence (EVA) Study – was designed to address several gaps in the literature. Firstly, we employed a bio-psycho-social framework with a comprehensive assessment battery at baseline to capture different aspects of functioning and everyday life experiences typical for this age group. Secondly, we used a longitudinal design to help disentangle the direction of effects between variables. The study primarily aimed to examine, *prospectively,* which and to what extent the wide range of biopsychosocial factors included will predict psychological distress (symptoms of depression and anxiety) and well-being in adolescents. Thirdly, the study sought to consider the predictive effects of these vulnerability markers on the developmental trajectory of depression and anxiety symptoms across the sensitive period of the teenage years. To this end, the target age range was set to cover the period immediately prior to the typical onset age of adolescent depression (see below for details). This is in recognition that early detection of these symptoms, and before illness onset, will help develop preventative and early intervention strategies. Finally, we chose to recruit participants from a range of state-funded and fee-paying secondary schools to achieve a representative sample of adolescents. It was a deliberate effort to recruit from the community, as research has shown that a large proportion of adolescents with psychological distress do not present in clinics ( Potrebny *et al.*, 2021); thus, a clinical sample would not represent the full spectrum of the depressive and anxiety symptoms in this population.

We hope that the findings from this longitudinal study will help identify biopsychosocial markers that could potentially be developed into screening tools to advance the clinical goal of earlier detection of symptoms, in contrast to the current diagnostic approach that is heavily reliant on subjective clinical judgement. Successful interventions at this critical developmental stage will help alleviate immediate suffering and have the potential to remedy perturbations of illness development, improving quality of life in the long run.

The current paper will focus on reporting data collected at the baseline phase. The objectives of the present report were two-fold. Firstly, it sought to provide a comprehensive overview of this longitudinal study, providing information on its background and methodology, as well as to summarise the demographic, clinical and mechanistic characteristics of the recruited sample at baseline. Secondly, this paper will present the distributions of the baseline measures in the study, providing descriptive statistics and examining individual differences in demographic, clinical and mechanistic measures among the adolescent sample by virtue of age, gender, and personal and family mental health history. This report is the first of a series of papers in which we will report hypothesis-driven analyses to address research questions concerning risk and resilience to adolescent depressive and anxiety symptoms.

## 2. Methods

### 2.1. Study design

The EVA study is a longitudinal study with data collection across three-time points. The baseline phase (Phase 1) was conducted across 13 months in 2018-2019, with recruitment spanning across 13 months, during which a sample of adolescents was recruited and asked to complete a comprehensive assessment protocol. The first follow-up (Phase 2) was conducted six months after the baseline phase in 2019, during which participants were contacted to complete three outcome measures assessing levels of depression, anxiety and well-being using the Online Survey platform (formerly known as Bristol Online Survey). We originally planned to contact the participants again for a final follow-up 12 months after the first follow-up. However, COVID and other circumstances caused delays, leading to the final follow-up taking place 60 months after the baseline (Phase 3, 2023). During this final follow-up, adolescents were asked to complete the same outcome measures of depression, anxiety and well-being, as well as a small subset of baseline measures selected based on preliminary analyses of the data collected in Phase 1.

### 2.2. Participants

A total of 425 adolescents between the ages of 12 and 18 ( *Mean age* = 15.06, *SD* = 1.75) were recruited from 12 schools in four council areas in Scotland (Edinburgh, East Lothian, Midlothian, and Kinross), UK, including both state-funded and independent (fee-paying) schools. The recruitment strategy was designed to cover as wide a geographical area as was practically feasible, with schools selected to reflect a broad range of socio-economic and educational contexts. Schools were initially approached via email, followed by telephone contact and in-person meetings, where feasible. Within participating schools, students were invited to take part through classroom presentations, flyers, and parental information packs.

The sample comprised 60.5% females ( *Mean age* =15.10, *SD* =1.72) and 34.4% males ( *Mean age* =14.86, *SD* =1.80). This age range was chosen to cover the years immediately prior to and around the typical onset age for adolescent depression and anxiety (i.e., ~15 years; Kessler *et al.*, 2007; Lewinsohn *et al.*, 1998); the relatively wide age range was deemed necessary to examine if age may be a factor that interacts with vulnerability traits. Demographic details are reported in full in Section 3.1. below.

### 2.3. Study procedure

Ethical approval was obtained from the Research Ethics Committee at the University of Edinburgh (Reference no. STAFF115) and the relevant local educational councils. When the study was moved to the University of Reading, further ethics approval was obtained from the University of Reading (Reference no. UREC 23_22). In Phase 1 ( *baseline*), potential participants met individually with one of the researchers in a private space at their school. During these face-to-face meetings, participants were seen alone to ensure confidentiality and to encourage open engagement. The researcher provided each participant with an information sheet to explain the overall rationale, procedure, and ethical considerations, and answer questions regarding the research study. Participants over the age of 16 years were asked to provide written informed consent for themselves; participants under the age of 16 years were asked to sign an assent form in addition to returning a parents’/guardians’ consent form. Participants were also asked to provide their email addresses and /or phone numbers to receive reminders regarding completing the online tasks and to be contacted for future follow-up studies. 

Further, participants were asked to provide demographic and background information and complete a comprehensive battery of emotional, cognitive, lifestyle, and biological measures. Details of the measures are provided in Section 2.4 below. As an overview, the assessment battery involved various face-to-face tasks followed by standardised age-appropriate online questionnaires. The full assessment session, including breaks, typically lasted between 45 and 60 minutes.

All the data collected from participants were stored against a unique identification code assigned to each participant and were used for completing the online measures during baseline and follow-up studies. The online measures were completed via the Online Survey (formally known as the Bristol Online Survey tool). Participants were free to complete the online tasks/measures at their own pace, time, and place to minimise the burden and impact of research on their daily activities. After completing the tasks and questionnaires, participants were presented with a full debrief page, including information signposting them to the organisations they could contact to seek mental health support. Participants were reimbursed with a £10 retail voucher for their participation.

### 2.4. Measures

Baseline assessments were completed in two stages. In a face-to-face meeting with the researcher, participants completed three outcome measures to index their current psychological distress (depression and anxiety) and well-being, as well as providing a hair sample for cortisol measure and obtained an Actiwatch for an over-night assessment of sleep quality (see 2.4.1 – 2.4.5). After the face-to-face session, participants completed online, age-appropriate and standardized questionnaires to assess a range of bio-psycho-social factors hypothesized to be associated with adolescents’ depression and anxiety (see 2.4.6 – 2.4.18). See Table 1 for a summary of the measures used. Internal reliability was further computed and verified to indicate good reliability in the current sample with the Cronbach’s alphas reported below (see Table 1).

**Table 1.** Summary of the Measures used and the Cronbach alpha’s in the current sample at baseline.

Factors	Measures	Cronbach Alpha’s
Outcome Measures		
Symptoms of Depression	Short Mood and Feelings Questionnaire (SMFQ; Angold *et al.*, 1995),	0.89
Symptoms of Anxiety	Generalised Anxiety Disorder-7 (GAD-7; Spitzer *et al.*, 2006).	0.87
Well-being	Short Warwick Edinburgh Mental Well Being Scale (SWEMWBS).	0.81
Biological		
Hair cortisol	Cortisol concentration within hair sample	N/A
Quality of sleep	Philips Actigraph Watch-2	N/A
Health and Lifestyle		
Eating Habits	Adolescent Food Habit Checklist (AFHC-23; Johnson *et al.*, 2002)	0.84
Eating Disorder Risk	Eating Attitudes Test ( Garner *et al.*, 1982)	0.90
Personality, Stress and Coping		
Resilience	Brief Resilience Scale (BRS; Smith *et al.*, 2008)	0.83
Stress	Perceived Stress Scale ( Cohen *et al.*, 1983)	0.88
Neuroticism	Eysenck's Short Neuroticism Scale ( Eysenck *et al.*, 1985).	0.79
Social and Interpersonal		
Others as Shamer	Other as Shamer ( Goss *et al.*, 1994)	0.95
Level of Emotional Support	Level of Expressed Emotions ( Cole & Kazarian, 1988)	0.93
Bullying and Cyberbullying Experiences	Traditional Bullying and Cyberbullying ( Hinduja & Patchin, 2010)	0.82 - 0.89
Cognitive		
Rumination	Ruminative Response Scale (RSR; Treynor *et al.*, 2003)	0.85
Dysfunctional Attitudes	Dysfunctional Attitudes Scale (DAS-24)	0.88
Attributional Bias	Short Form of the Ambiguous Scenarios Test for Depression in Adolescents (Short-AST-DA; Orchard *et al.*, 2018)	0.76
Self-Referential Effect	Self-Reference Categorisation and Recall Tasks ( Kelvin *et al.*, 1999)	--

***2.4.1. Symptoms of depression*.** The short version of the *Mood and Feelings Questionnaire* (SMFQ; Angold *et al.*, 1995), comprising 13 items based on the DSM criteria, was employed to assess depressive symptomatology among adolescents. The SMFQ is a reliable and valid measure developed for children and adolescents between 8 and 18 years ( Angold *et al.*, 1995). This measure assesses participants' feelings in the past two weeks on a three-point Likert scale, where *‘0’* refers to *‘not true’* and *‘2’* corresponds to *‘true’.* The total score ranges between 0 and 26, with participants scoring 12 or higher indicating the presence of depressive symptoms. The scale has been reported to have strong psychometric properties with an internal consistency between 0.91 and 0.95 ( Daviss *et al.*, 2006; Thabrew *et al.*, 2018).

***2.4.2. Symptoms of anxiety*.** The level of anxiety symptoms was assessed using a self-report 7-item *Generalised Anxiety Disorder Screener* (GAD-7; Spitzer *et al.*, 2006). The present measure has been normed with adolescents (from 14 years old) and has been widely used with children as young as 11. This scale assesses participants' severity of anxiety symptoms in the past two weeks on a four-point Likert scale ranging from *'not at all'* to *'nearly every day'*, with higher scores showing greater anxiety symptoms among participants. A scale score of 5, 10 and 15 has been recommended to indicate mild, moderate, and severe anxiety, respectively. The measure has been shown to have excellent psychometric properties in previous studies, with an internal consistency of 0.92 ( Spitzer *et al.*, 2006).

***2.4.3. Well-being*.** Participants’ well-being levels were assessed using a 7-item, self-report *Short Warwick-Edinburgh Mental Well-being Scale* (SWEMWBS). The scale comprises seven positively worded statements to assess participants' thoughts and feelings in the past two weeks on a five-point Likert scale, with *'1'* corresponding to *'none of the time'* and *'5'* meaning *'all the time'*, with higher total scores reflecting more positive well-being. The criteria for cut-offs at one standard deviation above or below the mean scores demarcate the thresholds for low, normal, and high well-being scores respectively. The scale has shown good psychometric properties, with reliability coefficients between 0.80 and 0.88 ( Fat *et al.*, 2017; Koushede *et al.*, 2019; McKay & Andretta, 2017; Ringdal *et al.*, 2018).

***2.4.4. Hair cortisol*.** Unlike salivary cortisol level which only indicated cortisol level at a single time point, longer-term cortisol exposure can be assessed through measurements of cortisol concentration within the hair samples (the longer the hair, the longer period of time captured). The cortisol hormone is stored within hair following blood flow through the skin. The hair grows approximately one cm per month; therefore, the one cm section closest to the scalp represents the cortisol concentration in the past month ( Wright *et al.*, 2015). Thus, the present study used a hair sample as a retrospective measure of longer-term cortisol exposure. Similar to previous research (e.g., Sauvé *et al.*, 2007), a hair sample, approximately one cm in diameter, was cut close to the scalp in the posterior vortex area of the head. The posterior vortex areas have been shown to demonstrate minimum variations in cortisol, providing reliable cortisol levels ( Sauvé *et al.*, 2007). The hair samples were tied in elastic bands, wrapped in tin foils and stored at -20 °C against the participant's unique code.

***2.4.5. Quality of sleep*.** To assess sleep quality, participants were provided with a Philips Actigraph Watch-2 to take away for one night. The researcher explained the process of wearing the actigraph on a non-dominant wrist – an unobtrusive wristband– for one night to assess their quality of sleep. The recorded variables included the number of sleep intervals, sleep duration, sleep onset latency (the time taken to fall asleep), sleep efficiency (the duration of sleep during the resting period), and Wake after sleep onset (WASO, the number of awakenings after sleep onset). The concept of sleep intervals refers to the total duration of uninterrupted sleep experienced by participants during one night. For instance, if an individual sleeps continuously for 8 hours, they would have one sleep interval. However, if their sleep is fragmented with intermittent wakefulness, multiple sleep intervals would be observed.

The actigraph watch comprises accelerometers to measure movement and environmental light sensors and skin temperature to estimate the sleep-wake cycle accurately ( Albu *et al.*, 2019). The actigraph watch has been widely used as a reliable and valid method to assess the sleep-wake cycle ( Rodriguez *et al.*, 2016).

In our investigation, we adopt a nuanced approach to measuring sleep efficiency. Traditionally, sleep efficiency is defined as the percentage of time spent asleep while attempting to sleep. However, our methodology diverges from this standard. Instead of focusing solely on percentages, we quantify sleep efficiency by measuring the actual amount of sleep obtained during the designated rest period, expressed in minutes. This novel approach enables us to provide a more precise and direct assessment of sleep quality and effectiveness within the designated timeframe. By accurately capturing the duration of restorative sleep, our methodology enhances our understanding of individual sleep patterns. This nuanced perspective is invaluable for unravelling the complexities of sleep behaviour and identifying factors that influence overall sleep quality.

***2.4.6. Demographic and background health and lifestyle factors*.** A set of demographic and background questions were asked to record the participant’s age, gender, BMI (height/ weight), ethnic background, personal and family mental health history, medication history, and lifestyle factors including social media usage, physical activity, and risky behaviour. Ethnicity was assessed using a question structure based on the methodology employed in the Generation Scotland Study ( Smith *et al.*, 2013). Participant self-identified their ethnic background using predefined categories aligned with UK national census classifications. Furthermore, family and personal mental health history was assessed through two self-reported items to determine whether the participant or any of their family members had ever received a diagnosis or sought help for mental health difficulty. The items were designed to capture both formally diagnosed conditions and informal help-seeking behaviours, allowing for a broader representation of mental health experiences.

Social media usage was assessed through multi-part self-report questions. Participants reported their average daily duration of social media use on school and non-school days, and the platforms most frequently used. The demographic section also collates information on participant’s self-reported emotional impact associated with their social media use (i.e., positive, negative, mixed, or neutral feelings).

For physical activity, participants were classified as ‘active’ if their engagement occurred two or more times per week (frequency) and extended for a minimum of two hours per week (duration; Centre for Disease Control and Prevention, 2021). In the present study, physical activity was defined as any activity that increases heart rate and may result in breathlessness. This encompasses various forms such as sports, school activities, playing with friends, or walking to school. Other example of physical activities included running, walking, cycling, dancing, skateboarding, swimming, football and gymnastics. Furthermore, a set of 11 questions from the Health and Behaviour in School Children survey (HBSC; Currie & Aleman-Diaz, 2015) was utilized to assess participants' risk behaviours, including the frequency and amount of smoking, alcohol consumption, and cannabis intake.

***2.4.7. Eating habits*.** *Adolescents Food Habits Checklist* (AFHC-23; Johnson *et al.*, 2002) was employed to assess participants' healthy and unhealthy eating behaviours. The scale comprised of 23 items to be rated as *'True', 'False'* or *'Not applicable'*. These items tap into the participant's food intake and dietary habits, such as consumption, purchasing, preparing, and snacking habits. The scores were computed by providing 1 point for all healthy responses, with higher scores showing healthier eating habits. The original psychometric analysis has demonstrated good internal consistency in an adolescent sample, α = 0.83 ( Johnson *et al.*, 2002).

***2.4.8. Eating disorder risk*.** A 26-item *Eating Attitudes Test* ( Garner *et al.*, 1982) was used to assess the risk of eating disorders based on participants' food-related attitudes, feelings, and behaviours. The items are rated on a 6-point Likert scale ranging from *‘Always’* to *‘Never’* (i.e., *always = 3*, *usually = 2*, *often = 1*, and *sometimes, rarely, and never = 0*). The total scores were computed by summing scores on all the items, with higher scores suggesting a greater concern for an Eating disorder risk. A scaled score of 20 or above shows a clinical concern about dieting, body weight or problematic eating behaviours among participants. The scale has been widely used as a highly reliable and valid measure in previous research, with internal consistency ranging between 0.86 and 0.90 ( Garner *et al.*, 1982).

***2.4.9. Resilience*.** A 6-item self-reported questionnaire *Brief Resilience Scale* (BRS; Smith *et al.*, 2008) was used to assess participant's ability to recover from stress. The scale comprises positively and negatively worded statements to rate at a five-point Likert scale, ranging from *'strongly disagree'* to *'strongly agree'*. The total scores are computed by summing the scores on all items and computing an average score by the number of questions answered. Higher total scores suggest higher resilience and a better ability to bounce back when experiencing stress ( Smith *et al.*, 2008). The scale has shown strong reliability and validity properties with internal consistency between 0.81 and 0.91 ( Smith *et al.*, 2008).

***2.4.10. Stress*.** The *Perceived Stress Scale* ( Cohen *et al.*, 1983), constituting 10 items, was used to measure participants' perception of unpredictable, uncontrollable, and overloading experiences in the past month. The PSS-10 measure has been widely used in previous research with young people and adults aged 12 and above. The items to assess stress levels are rated on a five-point Likert scale, with *'0'* corresponding to *'never'* and *'4'* meaning *'very often'*, with higher total scores indicating higher perceived stress. The scale has demonstrated acceptable to excellent internal consistency in previous students and adolescent samples, with α ranging between 0.73 and 0.91 ( Kechter *et al.*, 2019; Lee, 2012).

***2.4.11. Neuroticism*.** Neuroticism was measured using the self-report shortened form of the neuroticism subscale (12 items) from the *Eysenck Personality Questionnaire* ( Eysenck *et al.*, 1985). The items are rated on binary responses of *'Yes'* or *'No',* with each dichotomous item scored *1* or *0,* respectively. A higher total score corresponds to a higher neurotic trait. The scale is a widely employed, reliable, and valid measure, with previous studies showing good internal consistency of 0.84-0.88 ( Eysenck *et al.*, 1985; Smith *et al.*, 2018).

***2.4.12. Other as shamer*.** A self-reported *Other As Shamer Scale* ( Goss *et al.*, 1994) was used to assess participants' external shame, defined as one’s own perceptions of how others judge them. The measure comprises of 18-items rated on a five-point Likert scale ranging from *0 (Never)* to *4 (Almost always)*. Higher total scores reflect a higher tendency to perceive being judged negatively by others. The scale has shown excellent reliability coefficients in previous empirical studies, with the Cronbach's alpha ranging between 0.89 to 0.96 ( Goss *et al.*, 1994).

***2.4.13. Level of emotional support*.** A 38-item *Level of Expressed Emotion Questionnaire* ( Cole & Kazarian, 1988) was employed to measure participants’ perceived emotional support in their influential relationships. The items are rated on a four-point Likert scale, with ' *1'* meaning *'untrue'* and *'4'* suggesting *'true'*. Higher total scores represent a higher perceived lack of emotional support received from parents/guardians. The scale has shown a good internal consistency of 0.88 in a previous adolescent sample ( Nelis *et al.*, 2011).

***2.4.14. Bullying and cyberbullying experiences*.** Participants’ experience of bullying offending and victimisation was assessed using a 34-item *Traditional Bullying and Cyberbullying* ( Hinduja & Patchin, 2010) questionnaire that includes both traditional and cyber-bullying experiences. The items were related to individuals' experiences in school, friends, or family environments in the past 30 days. The items were rated on a five-point Likert scale ranging from *'Never (0)'* to *'Everyday (4)'*, and separate total scores were computed for each category, i.e., Bullying Offending, Bullying Victimisation, Cyberbullying offending, and Cyberbullying victimisation. The scale has shown satisfactory psychometric properties with Cronbach's alpha between 0.74 and 0.88 for the four categories ( Hinduja & Patchin, 2010).

***2.4.15. Rumination*.** A widely used, valid and reliable self-reported *Ruminative Response Scale* – short version (RSR; Treynor *et al.*, 2003) comprising 10 items was used to assess participants’ tendency to ruminate on two dimensions, i.e., brooding (self-criticism and negative evaluation) and reflection (problem-solving thoughts to overcome stress). The items are rated on a five-point Likert scale with *' 1 = almost never'* and *'5 = almost always'*. The total score ranges between 10 and 40, with higher scores reflecting a higher ruminative response style. The scale has shown good internal consistency in a previous adolescent sample (α = 0.85; Xavier *et al.*, 2016).

***2.4.16. Dysfunctional attitudes*.** A 24-item self-report *Dysfunctional Attitudes Scale* (DAS-24) was used to measure participants' dysfunctional beliefs and attitudes. The items were rated on a seven-point Likert scale ranging from ' *Totally agree =1'* to ' *Totally disagree =7',* and higher total scores indicated more dysfunctional attitudes. The scale has been widely employed as a reliable and valid measure with an internal consistency of 0.85 in previous adolescent samples ( Smith *et al.*, 2018).

***2.4.17. Attributional bias*.** The *Short Form of the Ambiguous Scenarios Test for Depression in Adolescents* (Short-AST-DA; Orchard *et al.*, 2018) was used to measure attributional bias. Participants were presented with 9 hypothetical ambiguous scenarios (For example, *"You go to a place you visited as a child. Walking around makes you emotional"*.) and asked to imagine each scenario as happening to them. Participants were asked to describe what they imagined and rated their imagined outcome on a 9-point scale ( *'1'= Not at all pleasant to ‘9'=Very pleasant*). Descriptive responses were coded as positive, negative, neutral, or mixed (both positive and negative) depending on the emotional content of the response. Two independent reviewers completed the coding process, and good inter-rater reliability was found between the two raters (Cohen's kappa = 0.80). The overall bias was calculated by subtracting the number of negative responses from the positive response. Thus, positive and negative scores represent positive and negative bias, respectively. The measure has shown acceptable internal consistency of 0.75 in previous research ( Orchard *et al.*, 2018).

***2.4.18. Self-Referential effect*.** *Self-Reference Categorisation and Recall Tasks* ( Kelvin *et al.*, 1999) were used to investigate participants’ biases in processing and remembering self-referenced information. The tasks involved the presentation of 15 positive (e.g., Skilful) and 15 negative adjectives (e.g., Worthless) where they first had to rate how *‘like me’* each descriptor is on a four-point scale ( *1= not at all,* to *4=very much*). Afterwards, participants were given an unexpected recall task, where they had to recall as many descriptors as possible. Response times, ratings, and recalled words were recorded.

Participants' ratings were recorded as either *'non-self-referent (not me)'* or *'self-referent (like me)'.* Similar to previous research, a proportional score reflecting overall positive bias in both 'me' and 'not me' conditions was calculated ( Smith *et al.*, 2018). The proportional scores were computed by subtracting the correctly recalled number of negative words from the correctly recalled number of positive words and dividing this by the total number of correctly recalled words. Thus, the final positive or negative scores represent a positive or negative bias, respectively.

### 2.5. Data analysis

Statistical analyses were conducted using IBM Statistics version 27 (Statistical packages for Social Sciences). Prior to the analyses, distributions, skewness, and kurtosis were assessed for the raw data. The absolute indexes of skewness and kurtosis showed approximate univariate normality with none of the indexes above ± 3 and ± 10 for skewness and kurtosis, respectively, one recommended cut-off for extremely skewed and kurtotic data ( Kline, 2011). The hair cortisol concentrations were log-transformed to fit the lognormal distributions ( Adam & Kumari, 2009; Smith *et al.*, 2018). The raw data were further analysed for improbable values to identify extreme outliers. No significant outliers were detected while examining Box plots for the total scores of the main study variables. However, three participants were identified to have extreme cortisol concentrations (i.e., identified as outliers with cortisol concentrations of 1645.78, 1338.01, and 68.65 pg/mg, +/- 4SD above and below the mean), which were subsequently excluded from the main analysis involving hair cortisol concentrations ( Smith *et al.*, 2018).

For the present report, descriptive statistics were examined to identify the baseline characteristics of the study participants. Additionally, independent sample t-tests were carried out to identify the individual differences at baseline based on gender and personal and family mental health history of the participants. Pearson Product Moment correlations were conducted to assess the associations between age, mental health outcome variables (i.e., depression, anxiety, and well-being scores) as well as other hypothesised bio-psycho-social mechanistic variables.

## 3. Results

### 3.1. Descriptive statistics

**Figure 1.**
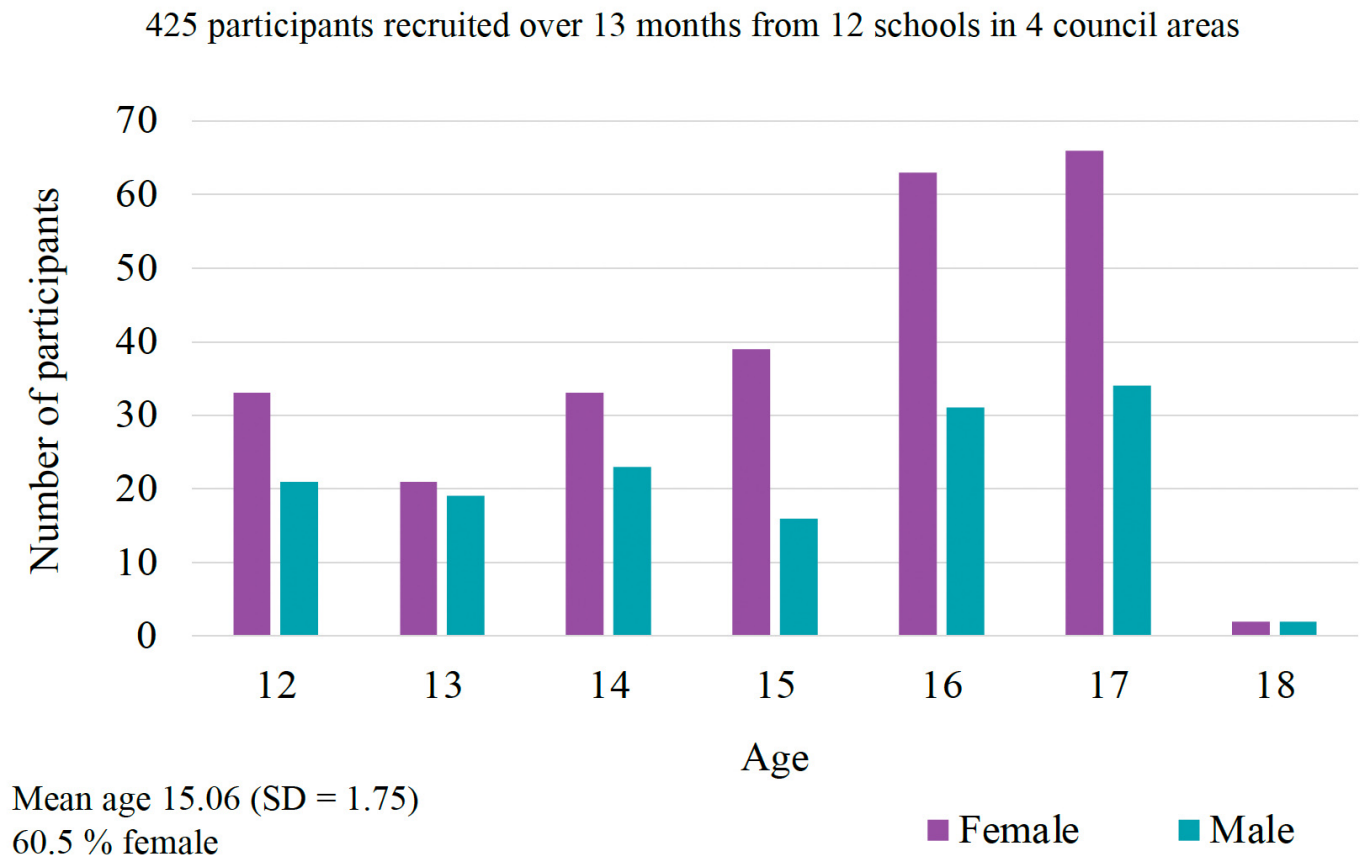
Age and gender distribution of EVA cohort.

***3.1.1. Baseline demographics and background health and lifestyle factors*.** The age distribution and details of the demographic characteristics are summarised in Figure 1 and Table 2. In brief, the majority of the sample was female adolescents (60.5%) and self-identified as being from a White ethnic background (70.6%). Half of the adolescents (49.9%) were within an ideal weight limit, defined by BMI's between 18.5 and 24.9 ( CDC, 2022). Nearly two-thirds (63.1%) of the sample reported no personal mental health history. However, a nearly equal percentage of participants reported the presence of a family mental health history (30.8%) compared to 34.4% of participants without any family mental health history (with the remaining third of the sample reporting ‘prefer not to say’, ‘don’t know’ or missing data). Participants' responses to the Health and Behaviour in School Children (HBSC; Currie & Aleman-Diaz, 2015) questions showed that the majority of adolescents do not smoke (71.3%) or use cannabis (75.5%), although 45% of adolescents reported alcohol intake ranging from occasionally to every once a week. Furthermore, a large proportion (40.5%) of our sample reported using 5 or more social media platforms, such as Facebook, Instagram, Pinterest, Snapchat, Tumblr, Twitter or YouTube and described positive feelings (44.5%) associated with using social media platforms. Additionally, nearly half of our sample (44.2%) reported being physically active two or more times a week for at least two or more hours per week (see Table 2).

**Table 2.** Demographic and Background Characteristics on Health and Mental Health, and Categorical Variables on Lifestyle Factors (N = 425).

Demographic Variable	Frequency	Percentage	Mean	Std. Deviation
Gender Males Females Other or non-binary Missing Data	146 257 2 20	34.4 60.5 0.5 4.7		
Age (12–18 years) Missing Data	420 5	98.8 1.2	15.05	1.753
Ethnicity White Non-White (Asian, Black, Mixed, Other) Missing Data	300 47 78	70.6 11.1 18.3		
BMI ^1^ Underweight Ideal Overweight Missing Data	15 212 24 174	3.5 49.9 5.6 40.9		
Personal Mental Health History No Yes Prefer not to say Don't know Missing Data	268 67 5 2 83	63.1 15.8 1.2 0.5 19.5		
Family Mental Health History No Yes Prefer not to say Don't know Missing Data	146 131 3 57 88	34.4 30.8 0.7 13.4 20.7		
Current Medication History ^2^ No Yes Prefer not to say Don't know Missing Data	288 44 6 3 84	67.8 10.4 1.4 0.7 19.8		
Current use of Psychotropic Drugs ^3^ No Yes Unsure	324 3 3	76.2 0.7 0.7		
Physically active ^4^ No Yes Missing Data	159 188 78	37.4 44.2 18.4		
Smokers Never Less than once a week At least one a week Everyday Missing Data	303 15 11 7 89	71.3 3.5 2.6 1.6 20.9		
Alcoholic Intake Never Hardly Ever Every month Every week Missing Data	152 71 91 30 81	35.6 16.7 21.4 7.1 19.1		
History of cannabis use Never Yes Missing Data	321 27 77	75.5 24.5 18.1		
Number of Social Media Platforms Used 1 2 3 4 5 6 7 >7 Missing data	13 22 63 79 110 44 14 4 76	3.1 5.2 14.8 18.6 25.9 10.4 3.3 0.9 17.9		
Feeling for using Social Media				
Positive	189	44.5		
Negative	21	4.9		
Mixed	30	7.1		
Neutral	110	25.9		
Missing data	74	17.4		
Time spent on Social Media during School days 15 min 45 min 75 min 105 min 2.5 hrs. 3.5 hrs. 4.5 hrs. 5.5 hrs. 6.5 hrs. Missing Values	35 52 61 48 43 52 25 21 10 78	8.2 12.2 14.4 11.3 10.1 12.2 5.9 4.9 2.4 18.4		
Time spent on Social Media during Non-School days 15 min 45 min 75 min 105 min 2.5 hrs. 3.5 hrs. 4.5 hrs. 5.5 hrs. 6.5 hrs. Missing Values	13 21 38 54 40 46 50 39 45 79	3.1 4.9 8.9 12.7 9.4 10.8 11.8 9.2 10.6 18.6		

^1^ BMI was calculated using https://www.stanfordchildrens.org/en/topic/default?id=childrens-bmi-calculator-41-ChildBMICalc; Underweight = less than 18.5; Ideal = 18.5-24.9; Overweight = 25.0-29.9. ^2^ Participants were asked if they were currently taking any medication. ^3^ Participants were asked to name the medication they are using currently. Medications names were checked by the research team to determine if they fall into the category of psychotropic drugs ^4^ ‘Physically active’= If participants stated that they were active 2 or more times a week (frequency) and for at least 2 hours a week (duration) in total they were coded as physically active.

***3.1.2. Baseline epidemiology*.** The summary of the adolescent sample providing usable data is provided in Table 3. The key outcome measures for depression, anxiety and well-being were successfully completed by 99.8%, 97.4% and 98.6% of adolescents, respectively. More than half of the sample provided biological samples for hair cortisol (60.7%) and sleep data (67.5%). Further, more than three-quarters of the sample provided data for health and lifestyle questions and completed social/interpersonal, personality, stress, coping, and cognitive measures.

**Table 3.** Summary of Phenotypes and samples available, and percentage providing valid/useable data.

Biopsychosocial Factors		Administration	Frequency	Percentage
**Demographics**	Age	Online	425	100%
	Gender	Online	405	95.3%
	Ethnicity	Online	346	81.4%
	Reported Mental History for self	Online	342	80.5%
	Reported Family's mental history	Online	337	79.3%
	Medication History	Online	341	80.2%
	Extracurricular Activities	Online	325	75.6%
	Physically Active	Online	347	81.6%
	Smoking History	Online	342	80.5%
	Alcohol History	Online	344	80.9%
	Drug History	Online	348	81.9%
**Health and Lifestyle**	BMI	Online	251	59.1%
	Social Media Usage	Online	350	82.4%
	Adolescent Food Habit Checklist	Online	339	79.8%
	Eating Attitudes Test	Online	325	76.5%
**Personality, Stress and Coping**	Brief Resilience Scale	Online	345	81.2%
	Perceived Stress Scale	Online	341	80.2%
	Eysenck's Short Neuroticism Scale	Online	340	80.0%
**Social and Interpersonal**	Other as Shamer	Online	337	79.3%
	Level of Expressed Emotions	Online	330	77.6%
	Traditional Bullying and Cyberbullying	Online	323	76.0%
**Biological**	Participants provided hair sample	Face-to-Face	262	61.6%
	Hair sample passed processing	Face-to-Face	255	60.0%
	Quality of Sleep (Sleep Efficiency)	Face-to-Face	287	67.5%
**Cognitive**	Ruminative Response Scale	Online	340	80.0%
	Dysfunctional Attitudes Scale	Online	334	78.8%
	Short Form of the Ambiguous Scenarios Test for Depression in Adolescents	Online	282	66.4%
	Self-Referential Effect	Online	331	77.9%
**Outcome Measures**	Short Mood and Feelings Questionnaire	Face-to-Face	424	99.8%
	Generalised Anxiety Disorder-7	Face-to-Face	414	97.4%
	Short Warwick Edinburgh Mental Well Being Scale	Face-to-Face	419	98.6%

The descriptive findings of the sample on measures of depression, anxiety, and well-being, as well as other measures of behavioural, cognitive, and social interpersonal variables, are presented in Table 4.

**Table 4.** Descriptive Results of Hypothesised Mechanistic Variables of the EVA Sample (N = 425).

Measure	N	Mean	Standard Deviation	Minimum	Maximum	Range	Theoretical Maximum Score Range
Eating Habits	339	11.89	5.11	0	23	23	0–23
Eating Disorder Risk	325	10.98	11.81	0.00	70.00	70	0–78
Resilience	345	3.05	0.75	1	5	4	1–5
Stress	341	20.17	7.93	0	40	40	10–40
Neuroticism	340	6.94	3.13	0	12	12	0–12
Other as Shamer	337	24.48	15.67	0	72	72	0–72
Level of Emotional support	330	78.90	18.23	46.22	134	87.78	38–152
Being a Bully	324	2.82	4.40	0	30.22	30.22	0–60
Being Bullied	323	6.78	9.21	0	55	55	0–76
Hair Cortisol Concentration	255	3.26	3.53	0.08	25.96	25.88	N/A
Quality of Sleep (Sleep efficiency)	287	84.38	6.98	61.10	96.56	35.46	N/A
Rumination	340	21.41	6.32	10	40	30	10–40
Attributional Bias	285	5.45	1.25	1.56	8.44	6.89	1–9
Self-Reference Bias	331	0.50	0.52	-1	1	2	0–12
Non-Self-reference Bias	331	-0.48	0.66	-1	1	2	0–12
Dysfunctional Attitudes	334	89.71	20.18	32.35	141	108.65	24–168
Depression	424	7.61	5.59	0.00	26.00	26	0–26
Anxiety	414	7.13	5.03	0	21	21	7–21
Well-Being	419	21.10	3.79	9.51	35	25.49	9.51–35

*Note.* Measures used to assess: Eating Habits = *Adolescents Food Habits Checklist*; Eating Disorder Risk = *Eating Attitudes Test*; Resilience = *Brief Resilience Scale;* Stress = *Perceived Stress Scale*; Neuroticism = *Eysenck Personality Questionnaire*; Others as Shamer = *Other As Shamer Scale*; Level of Emotional Support = *Level of Expressed Emotion Questionnaire*; Being a bully and bullied = *Traditional Bullying and Cyberbullying*; Rumination = *Ruminative Response Scale*; Attributional Bias = *Short Form of the Ambiguous Scenarios Test for Depression in Adolescents*; Self and Non-self-Reference Bias = *Self-Reference Categorization and Recall Task*s; Dysfunctional Attitudes = *Dysfunctional Attitudes Scale*; Depression = *Mood and Feelings Questionnaire*; Anxiety = *Generalised Anxiety Disorder Screener*; Well-being = *Short Warwick-Edinburgh Mental Well-being Scale*; Range = Highest value – Lowest value; Theoretical Maximum Score Range = provides the range of possible highest and lowest values; *Hair cortisol concentration* = level of cortisol hormone in pg/mg; *Sleep efficiency* = total minutes of sleep during the resting period.

***3.1.3. Clinical characteristics of the participants*.** Analyses on the self-reported measures of depression, anxiety, and eating attitudes suggest a broad range of symptom severity within the sample. While the majority of adolescents scored below clinical cut-offs, approximately 22% reported depressive symptoms above the clinical threshold, and only 36% fell within the normal range of anxiety. A smaller proportion (14.1%) were identified as at risk of developing an eating disorder. These findings suggest that although the overall sample includes many adolescents without clinical-level symptoms, a substantial minority are experiencing elevated psychological distress, consistent with community-based population trends (See Table 5).

**Table 5.** Clinical Characteristics of the Participants (N = 425).

Demographic Variable	Frequency	Percentage (%)	Means	Std. Deviation
Depression Level			7.61	5.59
Normal below cut-off	329	77.4		
Depressive, above cut-off	94	22.1		
Anxiety Level			7.13	5.03
Normal	152	35.8		
Mild	142	33.4		
Moderate	75	17.6		
Severe	45	10.6		
Eating Disorder Risk			10.98	11.81
Below cut-off score	265	62.4		
Above cut-off score	60	14.1		

*Note.* Measures used to assess Depression Level = *Mood and Feelings Questionnaire (SMFQ; Angold *et al.*, 1995)*; Anxiety Level = *Generalised Anxiety Disorder Screener ( Spitzer *et al.*, 2006)*; Eating Disorder Risk = *Eating Attitudes Test ( Garner *et al.*, 1982)*

### 3.2. Individual differences in baseline characteristics 

**Figure 2.**
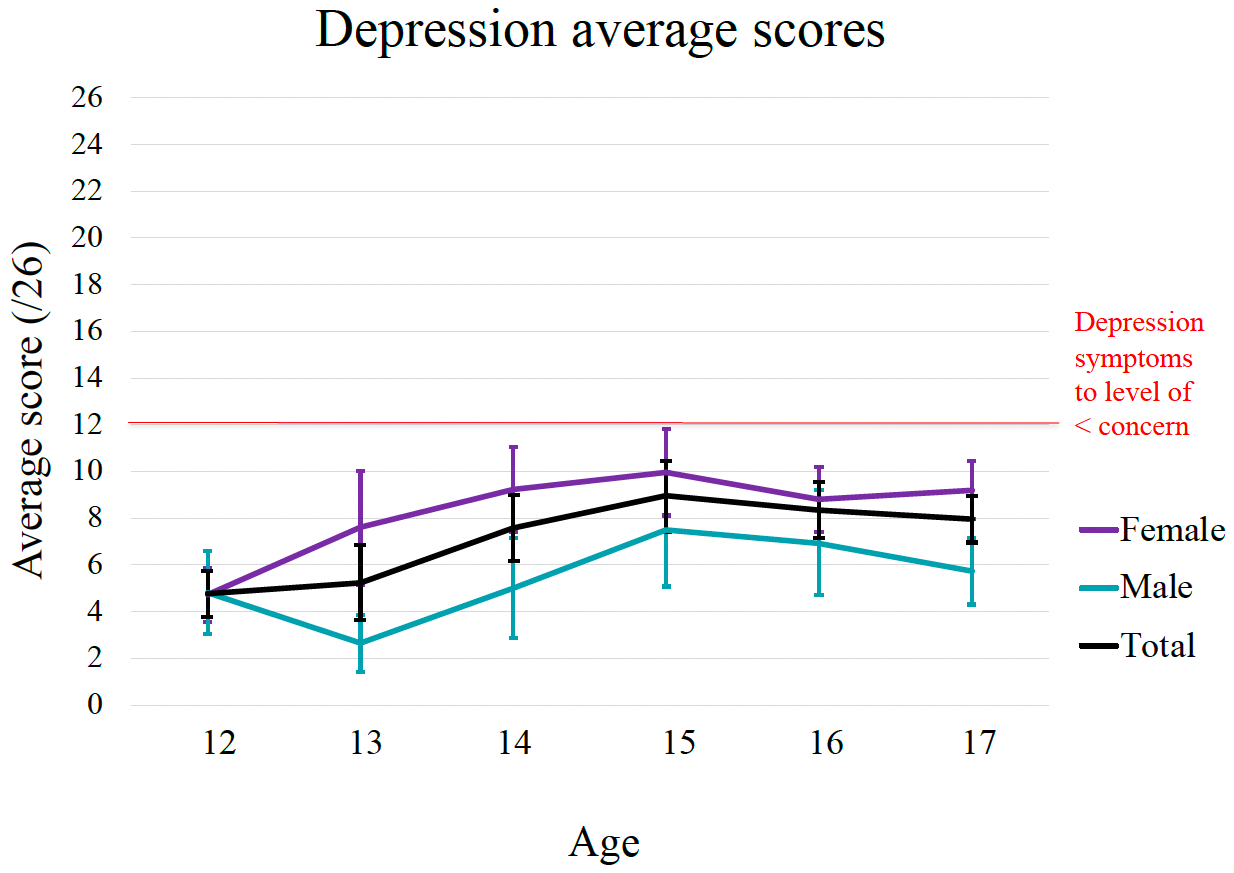
Age and gender differences in symptoms of depression for EVA cohort. The red line indicates the SMFQ clinical cut-off score of 12, above which symptoms may be considered indicative of depression.

***3.2.1. Age and gender differences on depression, anxiety and well-being*.** The age and gender differences in baseline levels of depression, anxiety and well-being are shown in Figure 2 – Figure 4. The graphical distribution of mean scores shows a general increase in depressive scores between 12 to 15 years which peaked around 15 years. Consistent with this, there was a statistically significant positive correlation between age with depression (r = 0.21, p < 0.001). Moreover, findings from the independent sample t-tests show statistically significant gender differences in depressive scores, t (400) = -5.49, p < 0.001, with females ( *M* = 8.53, *SD* = 5.48) reporting higher depressive scores compared to male adolescents ( *M* = 5.52, *SD* = 4.89). A Cohen’s d of 0.57 indicates a medium effect size.

**Figure 3.**
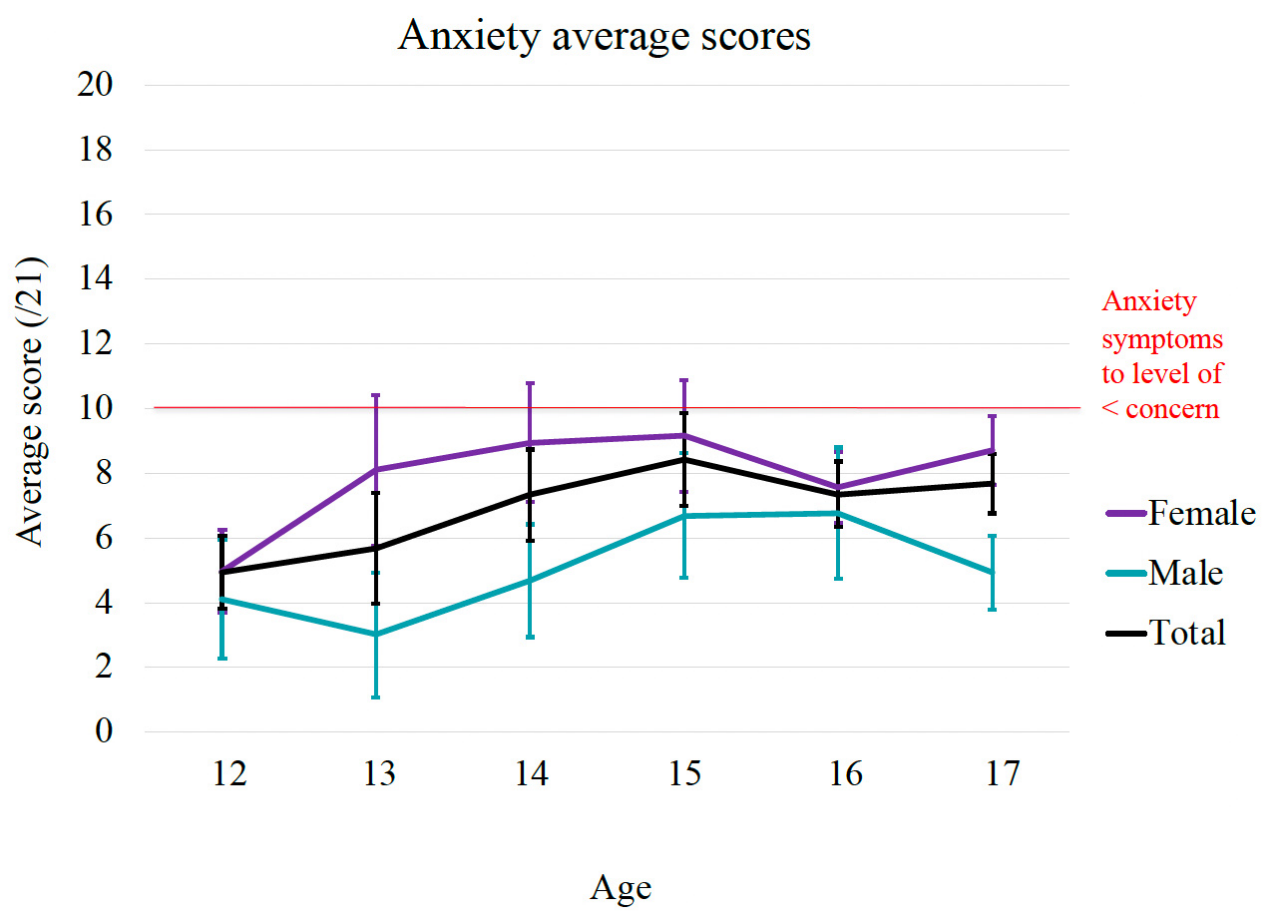
Age and gender differences in symptoms of anxiety. The red line represents the threshold score of 10, above which anxiety symptoms are considered to be at a level of concern.

**Figure 4.**
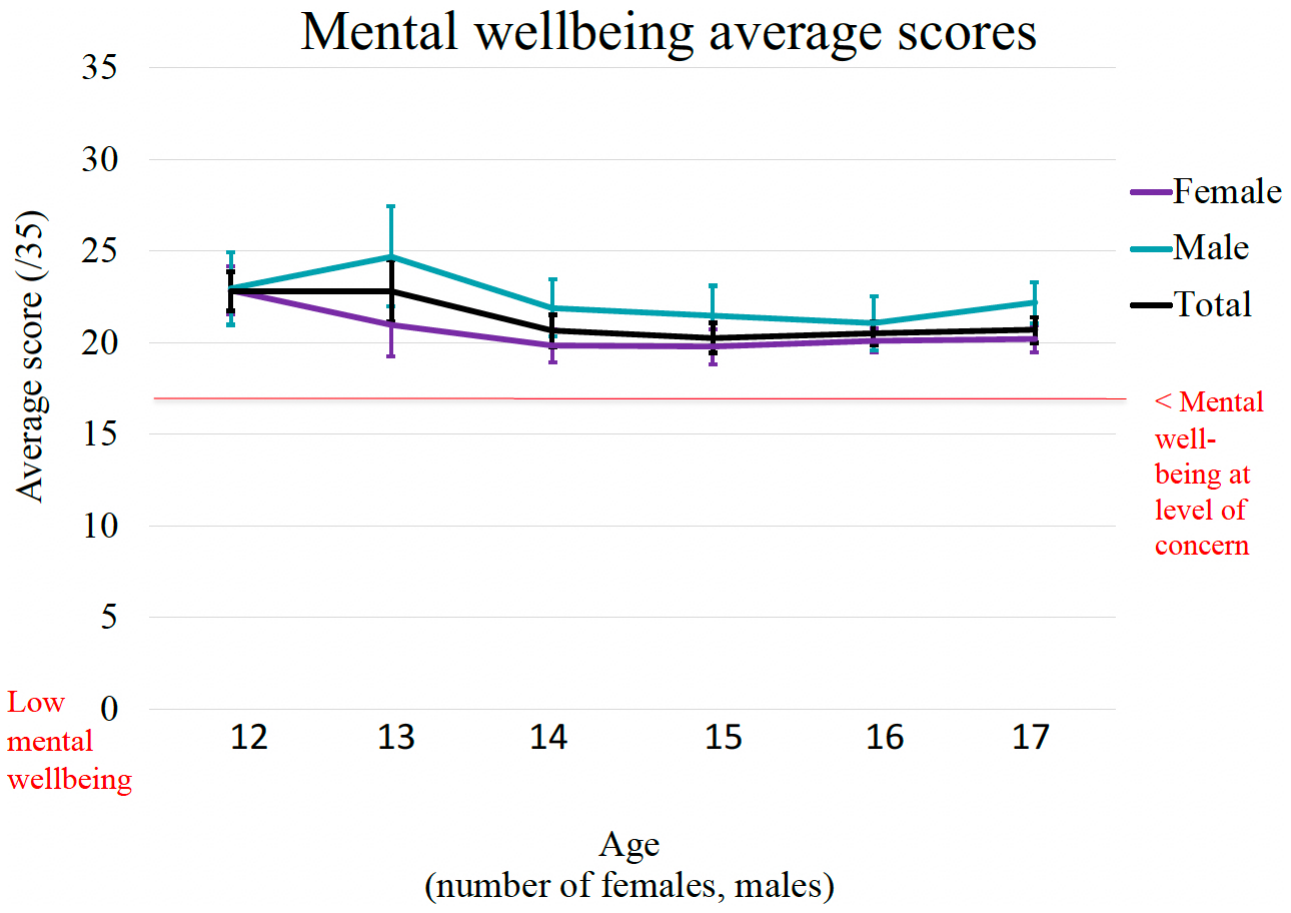
Age and gender differences in scores of well-being. Mental well-being at level of concern: SWEMWBS cut-off = 1 SD below sample mean.

Similarly, the graphical distribution of mean scores shows a general increase in anxiety scores between 12 to 15 years and peaked around 15 years of age (See Figure 3). Consistent with this, there was a statistically significant positive correlation between age and anxiety symptoms (r = 0.17, p < 000.1). Additionally, results from independent sample t-tests show a statistically significant gender difference in anxiety scores, t (391) = - 6.06, p < 0.001, driven by females ( *M* = 8.04, *SD* = 5.11) scoring higher average scores on anxiety measures compared to males ( *M* = 5.11, *SD* = 4.41). A Cohen's d of 0.62 signifies a medium effect size.

Finally, Figure 4 represents the age and gender distribution of the EVA sample for the Well-being scores. The graphical distribution of mean scores shows that well-being was generally higher among younger adolescents than older adolescents. Across the full age range of the sample, there was a statistically significant negative association between age and well-being (r = - 0.19, p < 000.1). Further, findings from the independent sample t-tests show statistically significant gender differences, t (395) = 4.46, p < 0.001, with males ( *M* = 22.29, *SD* = 4.30) having significantly higher well-being scores compared to females ( *M* = 20.47, *SD* = 3.14). A Cohen’s d of 0.50 indicates a medium effect size.

***3.2.3. Age and gender differences in biological variables*.** Correlational analysis and Independent sample t-tests were conducted to assess age and gender differences in biological variables of hair cortisol concentration and sleep quality. The correlational analysis suggests no significant associations between age and hair cortisol concentrations (r = 0.05, p = 0.39). However, there exist statistically significant associations between age and sleep duration (r = - 0.12, p < 0.05), sleep onset latency (r = 0.14, p < 0.05) and sleep efficiency (r = - 0.18, p < 0.01). These associations indicate that, with this age group, there is a tendency for older adolescents to experience reduced total sleep duration and to fall asleep earlier in comparison to their younger counterparts (See Table 6. For details). Further, the findings showed no statistically significant gender differences in hair cortisol concentrations, (t (241) = 1.77, p = 0.08). However, on the sleep quality variables, statistically significant differences were found in the number of sleep intervals, (t (273) = 2.36, p < 0.05), and sleep latency, (t (273) = -3.40, p < 0.001). These gender differences were driven by males reporting a greater number of sleep intervals while females reported taking longer to fall asleep. Effect size measures, however, indicated small effect sizes of these differences (d = 0.36 and 0.37 respectively).

**Table 6.** Age Difference in Study Variables (Pearson Product Moment Correlations).

Study Variables	Age
Eating Habits	-.18 **
Eating Disorder Risk	.14 *
Resilience	-.10
Stress	.30 **
Neuroticism	.22 **
Other as Shamer	.25 **
Level of Emotional support	.26 **
Being a bully	.22 **
Being Bullied	.06
Hair Cortisol Concentration	.05
Number of Sleep Intervals	-.12
Sleep Duration	-.12 *
Sleep Onset Latency	.14 *
Sleep Efficiency	-.18 **
Sleep Wakenings	.05
Rumination	.19 **
Attributional Bias	-.11
Self-referent bias	-.08
Non-self-reference bias	.14 **
Dysfunctional Attitudes	.23 **
Depression	.21 **
Anxiety	.17 **
Well-Being	-.19 **

*Note*. * significant at < 0.05; ** significant at < 0.01

***3.2.3. Age and gender difference in behavioural, cognitive and social/interpersonal variables*.** The correlational analysis indicates significant associations between age and hypothesized behavioural, cognitive, and social/interpersonal variables. Specifically, the results reveal a significant positive correlation between age and eating disorder risk, stress, neuroticism, other-as-shamer, level of emotional support, bullying offending, and dysfunctional attitudes. Conversely, a significant negative relationship is observed between age and eating habits (See Table 6. for the strength of associations). These findings suggest that, older adolescents tend to exhibit higher levels of eating disorder risk, stress, neuroticism, other-as-shamer tendencies, and dysfunctional attitudes. Additionally, they typically report higher levels of emotional support and engagement in bullying-offending behaviours. Interestingly, as age increases, there is likely a decrease in positive eating habits.

Gender differences in the hypothesised mechanistic variables were tested using independent sample t-tests. The findings are summarised in Table 7. The findings revealed significant differences, with medium to large Cohen’s d effect sizes, in measures of resilience, rumination, stress, neuroticism, others as shamer and being a bully. The findings showed that females reported a higher tendency to engage in ruminations, higher levels of stress and neuroticism and were more worried about how others saw them (i.e., higher external shame) than their male counterparts. On the contrary, males showed higher scores on resilience and were more likely to report being involved in bullying behaviours than females. See Table 6 for details. 

**Table 7.** Individual Difference in Study Variables based on Gender (Independent Sample t-tests).

Study Variables	Males		Females		t	p	Cohen's d
	Means	SD	Means	SD			
Eating Habits	11.22	5.15	12.22	5.10	-1.67	0.10	0.19
Eating Disorder Risk	7.35	7.31	12.60	13.03	-4.63	<.001 ***	0.46
Resilience	3.38	0.74	2.90	0.71	5.77	<.001 ***	0.67
Stress	16.29	7.80	21.97	7.30	-6.57	< .001 ***	0.76
Neuroticism	5.44	3.29	7.64	2.79	-6.04	< .001 ***	0.74
Other as Shamer	19.39	14.10	26.75	15.32	-4.14	< .001 ***	0.48
Level of Emotional support	79.32	17.92	78.78	18.45	0.25	0.80	0.03
Being a bully	4.06	5.23	2.27	3.85	3.12	0.002	0.41
Being Bullied	6.89	8.49	6.63	9.52	0.24	0.81	0.03
Hair Cortisol Concentration	2.62	3.43	3.38	3.00	-1.77	0.08	0.24
Number of Sleep Intervals	1.51	1.171	1.20	0.63	2.36	0.02 *	0.36
Sleep Duration	472.81	100.59	466.04	91.28	0.56	0.57	0.07
Sleep Onset Latency	18.33	21.32	30.14	36.27	-3.40	< .001 ***	0.37
Sleep Efficiency	84.76	7.09	84.33	6.90	0.50	0.62	0.06
Sleep Wakenings	36.26	20.89	31.67	18.36	1.87	0.06	0.24
Rumination	19.42	5.87	23.33	6.34	-4.02	< .001 ***	0.47
Attributional Bias	5.60	1.27	5.38	1.24	1.40	0.16	0.17
Self-referent bias	0.54	0.54	0.48	0.51	0.95	0.34	0.11
Non-self-reference bias	-0.60	0.58	-0.43	0.69	-2.29	0.23	0.26
Dysfunctional Attitudes	88.10	21.28	90.44	19.45	-0.99	0.32	0.12
Depression	5.52	4.89	8.53	5.48	-5.49	< .001 ***	0.57
Anxiety	5.11	4.41	8.04	4.87	-6.057	< .001 ***	0.62
Well-Being	22.29	4.30	20.47	3.14	4.46	< .001 ***	0.50

*Note*. *** significant at < 0.001, * significant at < 0.05; Measures used to assess: Eating Habits = *Adolescents Food Habits Checklist*; Eating Disorder Risk = *Eating Attitudes Test*; Resilience = *Brief Resilience Scale;* Stress = *Perceived Stress Scale*; Neuroticism = *Eysenck Personality Questionnaire*; Others as Shamer = *Other As Shamer Scale*; Level of Emotional Support = *Level of Expressed Emotion Questionnaire*; Being a bully and bullied = *Traditional Bullying and Cyberbullying*; Rumination = *Ruminative Response Scale*; Attributional Bias = *Short Form of the Ambiguous Scenarios Test for Depression in Adolescents*; Self and Non-self-Reference Bias = *Self-Reference Categorization and Recall Task*s; Dysfunctional Attitudes = *Dysfunctional Attitudes Scale*; Depression = *Mood and Feelings Questionnaire*; Anxiety = *Generalised Anxiety Disorder Screener*; Well-being = *Short Warwick-Edinburgh Mental Well-being Scale; Hair cortisol concentration* = level of cortisol hormone in pg/mg; *Sleep duration* = Total duration of sleep intervals in minutes; *Sleep onset latency* = minutes to fall asleep; *Sleep efficiency* = total minutes of sleep during the resting period; *Sleep Wakenings* = number of wakening events after falling asleep.

***3.2.4. Individual differences in the studied variables based on Personal Mental Health History*.** Independent sample t-tests were conducted to examine the differences in measures of depression, anxiety, well-being and other cognitive and behavioural measures based on the self-reported mental health history. A summary is provided in Table 8. The findings demonstrated that adolescents with self-reported mental health history had significantly higher current depressive and anxiety scores and lower well-being scores compared to adolescents without a mental health history, with large effect sizes.

**Table 8.** Individual Difference in Study Variables based on Personal Mental Health History (Independent Sample T-tests).

Study Variables	No		Yes		t	p	Cohen's d
	Means	SD	Means	SD			
Eating Habits	11.61	5.08	12.59	5.30	-1.38	.167	0.190
Eating Disorder Risk	9.51	10.52	16.02	14.60	-3.39	<.001 ***	0.57
Resilience	3.23	.69	2.39	.64	8.96	<.001 ***	1.226
Stress	18.41	7.56	26.89	5.23	-10.69	<.001 ***	1.185
Neuroticism	6.36	3.06	9.33	2.29	-8.73	<.001 ***	1.015
Other as Shamer	21.21	14.16	37.74	13.92	-8.54	<.001 ***	1.171
Level of Emotional support	77.74	17.74	85.94	18.52	-3.31	<.001 ***	0.458
Being a bully	2.93	4.48	2.67	4.46	.411	.681	0.057
Being Bullied	5.65	7.91	10.22	12.31	-3.66	<.001 ***	0.505
Hair Cortisol Concentration	3.12	3.53	3.56	3.18	-0.74	0.46	0.125
Number of Sleep Intervals	1.32	0.91	1.18	0.52	1.43	0.16	0.17
Sleep Duration	464.06	96.08	468.46	93.68	-0.29	0.77	0.05
Sleep Onset Latency	26.28	34.29	27.75	30.89	-0.28	0.78	0.04
Sleep Efficiency	84.44	7.24	84.11	6.94	-0.28	0.78	0.05
Sleep Wakenings	33.16	18.75	32.09	21.46	0.35	0.73	0.06
Rumination	20.32	6.02	25.84	5.77	-6.73	<.001 ***	0.923
Attributional Bias	5.59	1.22	4.84	1.26	4.08	<.001 ***	0.607
Self-referent bias	.55	.50	.31	.56	3.25	.002 **	0.480
Non-self-reference bias	-.56	.61	-.19	.76	-3.68	<.001 ***	0.574
Dysfunctional Attitudes	86.65	19.69	100.55	18.70	-5.16	<.001 ***	0.713
Depression	6.21	4.65	12.44	5.61	-8.39	<.001 ***	1.283
Anxiety	5.90	4.18	11.61	5.00	-8.54	<.001 ***	1.310
Well-Being	21.62	3.72	18.75	2.38	7.74	<.001 ***	0.820

*Note*. *** significant at < 0.001; ** at 0.01. Measures used to assess: Eating Habits = *Adolescents Food Habits Checklist*; Eating Disorder Risk = *Eating Attitudes Test*; Resilience = *Brief Resilience Scale;* Stress = *Perceived Stress Scale*; Neuroticism = *Eysenck Personality Questionnaire*; Others as Shamer = *Other As Shamer Scale*; Level of Emotional Support = *Level of Expressed Emotion Questionnaire*; Being a bully and bullied = *Traditional Bullying and Cyberbullying*; Rumination = *Ruminative Response Scale*; Attributional Bias = *Short Form of the Ambiguous Scenarios Test for Depression in Adolescents*; Self and Non-self-Reference Bias = *Self-Reference Categorization and Recall Task*s; Dysfunctional Attitudes = *Dysfunctional Attitudes Scale*; Depression = *Mood and Feelings Questionnaire*; Anxiety = *Generalised Anxiety Disorder Screener*; Well-being = *Short Warwick-Edinburgh Mental Well-being Scale*; *Hair cortisol concentration* = level of cortisol hormone in pg/mg; *Sleep duration* = Total duration of sleep intervals in minutes; *Sleep onset latency* = minutes to fall asleep; *Sleep efficiency* = total minutes of sleep during the resting period; *Sleep Wakenings* = number of wakening events after falling asleep.

The results further show that adolescents reporting a history of mental health issues have statistically significant and higher scores on rumination, stress, neuroticism, others as shamer, dysfunctional attitudes, lack of emotional support and greater bullying experiences. Conversely, adolescents without a mental health history have significantly higher scores on resilience, self-reference, non-self-reference and attributional bias. In other words, individuals who do not report a mental health history appeared to exhibit greater resilience and a greater tendency to perceive and interpret social situations more positively. They also appeared to be more likely to cultivate positive relationships and interactions with others. (See Table 8). Medium to Large effect sizes were noted for the above differences.

***3.2.5. Individual differences in the research variables based on Family Mental Health History*.** The individual differences based on the participants' family mental health history were also examined using an independent sample t-test. The results showed significantly higher depressive and anxiety scores and lower well-being scores among adolescents with a family history of mental health issues, with medium to large effect sizes.

Furthermore, similar to the previous results on personal mental health history, the findings show that adolescents with a family history of mental illness had significantly higher scores on measures of rumination, stress, neuroticism, others as shamer, dysfunctional attitudes, and had greater bullying victimisation. By contrast, adolescents without a family mental health illness were likely to have greater resilience and less attributional biases. Table 9. summarises the results of individual differences based on family mental health history across all the study variables. The above differences demonstrated medium to large effect sizes.

**Table 9.** Individual Difference in Study Variables based on Family's Mental Health History (Independent Sample t-tests).

Study Variables	No		Yes		t	p	Cohen's d
	Means	SD	Means	SD			
Eating Habits	12.02	5.17	12.06	5.14	-.06	.952	0.007
Eating Disorder Risk	9.25	9.89	13.20	13.40	-2.68	0.008	0.34
Resilience	3.30	.69	2.91	.77	4.41	<.001 ***	0.537
Stress	17.54	7.34	22.94	7.76	-5.88	<.001 ***	0.715
Neuroticism	6.05	3.06	7.77	3.01	-4.65	<.001 ***	0.568
Other as Shamer	19.30	13.06	29.74	16.28	-5.75	<.001 ***	0.711
Level of Emotional support	77.37	17.83	81.62	19.06	-1.87	0.063	0.231
Being a bully	2.72	4.32	3.17	5.06	-.78	0.439	0.097
Being Bullied	5.01	7.94	8.30	10.29	-2.86	.005 **	0.359
Hair Cortisol Concentration	3.17	3.78	3.10	2.80	0.15	0.88	0.022
Number of Sleep Intervals	1.37	0.98	1.25	0.81	0.91	0.36	0.13
Sleep Duration	467.16	93.80	471.95	95.61	-0.36	0.72	0.05
Sleep Onset Latency	26.03	34.69	29.21	35.78	-0.64	0.53	0.09
Sleep Efficiency	84.35	7.00	84.02	7.57	0.51	0.38	0.05
Sleep Wakenings	33.82	19.98	33.83	20.41	-0.01	0.99	0.001
Rumination	19.44	5.84	23.72	6.39	-5.75	<.001 ***	0.702
Attributional Bias	5.69	1.11	5.21	1.36	2.91	.004 **	0.381
Self-referent bias	.56	.53	.46	.53	1.60	0.110	0.197
Non-self-reference bias	-.67	.52	-.39	.71	-3.61	<.001 ***	0.450
Dysfunctional Attitudes	87.38	20.07	93.22	20.24	-2.36	.019 *	0.290
Depression	5.22	4.20	9.29	5.63	-6.76	<.001 ***	0.827
Anxiety	5.60	4.16	8.18	5.04	-4.58	<.001 ***	0.561
Well-Being	21.82	3.84	20.28	3.41	3.49	<.001 ***	0.422

*Note*. *** significant at < 0.001; ** at 0.01; * at 0.05. Measures used to assess: Eating Habits = *Adolescents Food Habits Checklist*; Eating Disorder Risk = *Eating Attitudes Test*; Resilience = *Brief Resilience Scale;* Stress = *Perceived Stress Scale*; Neuroticism = *Eysenck Personality Questionnaire*; Others as Shamer = *Other As Shamer Scale*; Level of Emotional Support = *Level of Expressed Emotion Questionnaire*; Being a bully and bullied = *Traditional Bullying and Cyberbullying*; Rumination = *Ruminative Response Scale*; Attributional Bias = *Short Form of the Ambiguous Scenarios Test for Depression in Adolescents*; Self and Non-self-Reference Bias = *Self-Reference Categorization and Recall Task*s; Dysfunctional Attitudes = *Dysfunctional Attitudes Scale*; Depression = *Mood and Feelings Questionnaire*; Anxiety = *Generalised Anxiety Disorder Screener*; Well-being = *Short Warwick-Edinburgh Mental Well-being Scale*; *Hair cortisol concentration* = level of cortisol hormone in pg/mg; *Sleep duration* = Total duration of sleep intervals in minutes; *Sleep onset latency* = minutes to fall asleep; *Sleep efficiency* = total minutes of sleep during the resting period; *Sleep Wakenings* = number of wakening events after falling asleep.

***3.2.6. Individual differences in the research variables based on Ethnicity (Independent sample t-test).*** Individual differences based on participants’ ethnicity were examined using independent samples t-tests. The results indicated no significant differences between White and non-White participants on any of the study variables. Table 10 summarises the results of these analyses across all research variables.

**Table 10.** Individual Difference in Study Variables based on Ethnicity (Independent Sample t-tests).

Study Variables	White		Non-White		t	p	Cohen's d
	Means	SD	Means	SD			
Eating Habits	11.99	5.15	11.22	4.92	0.94	0.35	0.15
Eating Disorder Risk	11.28	12.01	9.27	10.56	1.05	0.29	0.17
Resilience	3.06	0.76	3.01	0.68	0.43	0.67	0.07
Stress	20.07	8.01	20.50	7.61	-0.35	0.73	-0.06
Neuroticism	6.98	3.13	6.63	3.19	0.70	0.49	0.11
Other as Shamer	24.79	15.56	22.01	16.43	1.12	0.27	0.18
Level of Emotional support	78.46	18.55	81.23	16.35	-0.95	0.35	-0.15
Being a bully	2.89	4.55	2.47	3.42	0.59	0.55	0.10
Being Bullied	7.08	9.53	5.05	6.86	1.35	0.18	0.22
Hair Cortisol Concentration	18.27	149.38	4.52	4.44	0.45	0.65	0.10
Sleep Duration	465.02	96.76	472.81	87.16	-0.44	0.66	-0.08
Sleep Onset Latency	27.27	34.21	21.87	26.42	0.88	0.38	0.16
Sleep Efficiency	84.58	6.95	83.26	7.876	1.02	0.31	0.19
Rumination	21.39	6.29	21.07	6.51	0.32	0.75	0.05
Attributional Bias	5.44	1.24	5.46	1.29	-0.07	0.95	-0.01
Self-referent bias	0.487	0.53	0.60	0.41	-1.36	0.18	-0.22
Non-self-reference bias	-0.50	0.65	-0.40	0.72	-0.91	0.37	-0.15
Dysfunctional Attitudes	90.08	20.29	87.75	19.96	0.72	0.47	0.12
Depression	7.52	5.55	6.99	4.59	0.62	0.54	0.10
Anxiety	7.13	5.05	6.20	3.89	1.21	0.23	0.19
Well-Being	21.15	3.69	20.68	3.35	0.81	0.42	0.13

*Note*. *** significant at < 0.00 *Note*. *** significant at < 0.001; ** at 0.01. Measures used to assess: Eating Habits = *Adolescents Food Habits Checklist*; Eating Disorder Risk = *Eating Attitudes Test*; Resilience = *Brief Resilience Scale;* Stress = *Perceived Stress Scale*; Neuroticism = *Eysenck Personality Questionnaire*; Others as Shamer = *Other As Shamer Scale*; Level of Emotional Support = *Level of Expressed Emotion Questionnaire*; Being a bully and bullied = *Traditional Bullying and Cyberbullying*; Rumination = *Ruminative Response Scale*; Attributional Bias = *Short Form of the Ambiguous Scenarios Test for Depression in Adolescents*; Self and Non-self-Reference Bias = *Self-Reference Categorization and Recall Task*s; Dysfunctional Attitudes = *Dysfunctional Attitudes Scale*; Depression = *Mood and Feelings Questionnaire*; Anxiety = *Generalised Anxiety Disorder Screener*; Well-being = *Short Warwick-Edinburgh Mental Well-being Scale*; *Hair cortisol concentration* = level of cortisol hormone in pg/mg; *Sleep duration* = Total duration of sleep intervals in minutes; *Sleep onset latency* = minutes to fall asleep; *Sleep efficiency* = total minutes of sleep during the resting period; *Sleep Wakenings* = number of wakening events after falling aslee

## 4. Discussion

The present paper provides an overview of the EVA study, particularly highlighting the goal to assess vulnerability markers for adolescent depression and anxiety using a bio-psycho-social approach. This report also provides a comprehensive outline of the methodology, study design, and recruitment strategies used during the baseline phase of the longitudinal study. This report further summarised baseline characteristics of the participants and examined individual differences based on age, gender, and personal and family mental health history of the participants.

The key findings demonstrate that depressive and anxiety scores were significantly higher among adolescents between 15 and 18 years than the younger adolescents, with a peak around 15. These findings are broadly consistent with a recent meta-analysis of 192 epidemiological studies, which suggested that most mental health conditions begin by age 14, with the peak age of onset for depression and anxiety around 15.5 years ( Solmi *et al.*, 2022). Further, previous research has also highlighted that adolescents' depression and anxiety symptoms tend to start emerging during mid to late adolescence, leading to recurring mental health conditions in adulthood if left untreated ( Alaie *et al.*, 2023; Kessler *et al.*, 2005). Consistent with previous research, the findings from this cohort also revealed that females had significantly higher depressive and anxiety scores compared to male adolescents ( Morken *et al.*, 2023; Thapar *et al.*, 2012).

These research findings provide additional support for the need for mental health interventions during this vulnerable period and emphasise the need for targeted preventative and early intervention programmes in schools and communities ( Singh *et al.*, 2022; Wiedermann *et al.*, 2023). Aligned with previous research, findings support the need for mental health initiatives in schools to be improved to address the specific needs of adolescents from around the age of 15 ( Cavioni *et al.*, 2021). Besides, it is essential to have comprehensive and long-term monitoring and support systems in place, as most mental health conditions begin by age 14 and, if left untreated, are likely to persist into adulthood ( Cavioni *et al.*, 2021). Even within this sample, it was clear that personal history of mental health problems was associated with higher levels of psychological distress and lower levels of wellbeing. These findings therefore call for the integration of mental health concerns into broader public health policies ( Colizzi *et al.*, 2020) so that there would be greater coherence in the support we provide for adolescents through this challenging developmental stage. Related to this, a holistic approach to addressing the multifaceted origins of adolescent mental health challenges is crucial to prevent the potential long-term consequences of untreated conditions.

Likewise, it is important to note that mental health is considered essential to overall health and well-being ( WHO, 2022). The World Health Organisation defines health as a state of complete physical, mental, and social well-being beyond the absence of disease or infirmity ( WHO, 2022). In other terms, the recent definition by WHO emphasizes that mental health is not just about the absence of mental disorders or disabilities rather, it represents a state of well-being with an individual’s abilities to cope effectively with life's normal stresses, contribute productively to their communities, and ultimately enhance collective and individual capacities in daily living ( WHO, 2022). This holistic approach highlights the importance of evaluating and measuring well-being levels beyond addressing depression or anxiety. By assessing well-being, our study aimed to move beyond conventional research which tended to focus on psychiatric symptoms; instead we hoped to also identify biopsychosocial factors that contribute to an individual's ability to thrive, find fulfilment, and actively contribute to their community ( Ruggeri *et al.*, 2020). Furthermore, measuring well-being provides insights into individuals' strengths, resilience, and coping mechanisms, which sheds light on the aspects that enable them to navigate life's challenges successfully ( WHO, 2022).

In addition to showing a trend towards worsening symptoms of depression and anxiety over the teenage period, our findings also suggested a trend in lowering well-being scores with advancing age in adolescence, consistent with previous research ( Casas & González-Carrasco, 2019; Inchley *et al.*, 2016). Furthermore, our findings are consistent with existing literature demonstrating that girls reported lower levels of well-being ( Gómez-López *et al.*, 2019; Inchley *et al.*, 2016). Collectively, our findings and those of others propose that greater life satisfaction and well-being may serve as significant protective factors for adolescent mental health ( Kassis *et al.*, 2022; Patalay & Fitzsimons, 2018). These findings not only enhance our understanding of mental health but also provide direction for developing interventions that cultivate and amplify the inherent capacities for well-being within individuals and communities. Furthermore, these findings from the community-recruited EVA sample, where the distribution was inevitably skewed towards the lower end of depression and anxiety, provide particularly generalizable evidence relevant to adolescents whom we typically see outside clinics.

Furthermore, the results of this study reveal interesting patterns in a range of biopsychosocial variables as individuals progress through adolescence. Older adolescents exhibit elevated levels of several concerning factors, including being at higher risk for developing eating disorders. In line with previous findings, these results suggest that the onset of eating disorders often occurs during the later adolescent years ( Rohde *et al.*, 2015). Besides, the current findings replicate previous findings in proposing that older adolescents perceive greater levels of stress ( Wright *et al.*, 2023), neuroticism ( Aldinger *et al.*, 2014; Lahey, 2009), and dysfunctional attitudes. Notably, they also report greater engagement in bullying-offending behaviours, which warrants attention in intervention efforts ( Piquero *et al.*, 2016). Conversely, older adolescents appear to receive higher levels of emotional support, suggesting potential shifts in social dynamics or support networks as individuals mature ( Wickramaratne *et al.*, 2022). One particularly noteworthy finding is the apparent decline in positive eating habits with age, underscoring the importance of addressing dietary behaviours and promoting healthy lifestyles among adolescents ( Chaudhary *et al.*, 2020). These findings shed light onto the complex interplay between age and psychological variables during this critical developmental period, thus emphasising the need for targeted interventions to support adolescent well-being.

Additionally, the gender differences observed on the range of biological, behavioural, cognitive, social, and interpersonal factors assessed in this study are consistent with previous research. Of particular importance, the present findings showed that females were at greater risk of developing eating disorders, which might occur due to the interplay of transdiagnostic multifactorial factors that overlap with those predicting adolescents' depression and anxiety ( Batista *et al.*, 2018; Morris *et al.*, 2010). Furthermore, the current study replicated previous findings in suggesting that female adolescents had a greater tendency to ruminate about their negative moods ( Johnson & Whisman, 2013) and experience greater stress, neuroticism, and negative emotions in response to perceived threats ( Verma *et al.*, 2011; Weisberg *et al.*, 2011). Besides, the findings from the current cohort suggested that females are likely to have greater external shame and fear of being negatively judged. On the contrary, consistent with previous research, the findings showed that males have greater psychological resilience ( Gok *et al.*, 2024) and are more likely to be perpetrators of bullying behaviours. Gender differences in risk for depression and anxiety have been well documented; these findings extend the literature by providing further insights into the possible factors and mechanisms that may underscore these gender differences. They further highlight the importance of developing gender-tailored approaches that may improve the effect sizes of interventions

In terms of possible biomarkers of adolescent depression and anxiety, although no statistically significant gender differences were observed in hair cortisol concentrations, the significant differences in sleep quality parameters are noteworthy. Males exhibited a greater number of sleep intervals, suggesting possible differences in sleep patterns ( Okano *et al.*, 2019), while females took longer to fall asleep and experienced a delayed onset during the longest sleep interval ( Kabrita & Hajjar-Muça, 2016; Koikawa *et al.*, 2016). These results have implications for understanding the nuances of sleep-related behaviours and their potential associations with depression, anxiety, and well-being. Further research is warranted to explore the underlying mechanisms and potential implications of these gender-specific sleep patterns, which may inform targeted interventions and contribute to a more nuanced understanding of sleep health.

Our findings further replicate previous research findings in suggesting that adolescents with personal or family mental illnesses are likely to be three to four times more susceptible to developing depression or anxious symptoms ( Thapar *et al.*, 2012). Moreover, evidence from twin and family studies has largely proposed an increased inherited liability for early personal and family mental health illness in developing depression and anxiety during adolescence ( Thapar *et al.*, 2012). Consistent with this, previous research evidence supports current findings in proposing that adolescents without personal and mental illness history are likely to have greater well-being ( Alegria *et al.*, 2019; García-Carrión *et al.*, 2019) and resilience ( Mesman *et al.*, 2021), which ensures better life satisfaction ( Schlack *et al.*, 2021). Together, these findings propose that positive and negative personal as well as familial contexts play a crucial role in predicting mental health outcomes among adolescents ( Alegria *et al.*, 2019).

Similar individual differences in personal and family mental illness histories were observed on some of the bio-psycho-social factors assessed in the present study. The findings were consistent with a vast array of literature evidence in proposing that adolescents with previous personal and familial mental health crises are likely to have greater negative outcomes, such as stress; rumination ( Grierson *et al.*, 2016); neuroticism ( Lahey, 2009; Ormel *et al.*, 2013), dysfunctional attitudes, and bullying victimisation ( Moore *et al.*, 2017). On the basis of the baseline data reported in this paper, we cannot disentangle the direction of the effects. We will, however, examine these using the longitudinal data that are currently being collected as part of the EVA project.

### 4.1. Strengths and weaknesses

The EVA study has collected data on a wide range of biological, social, lifestyle/health, personality, coping and stress, and cognitive factors to examine vulnerability and resilience to adolescent depression, anxiety and well-being using a holistic approach. Considerable effort has been put into the recruitment process to maximise the representativeness of the sample, in particular by including adolescents from both state and fee-paying secondary schools across four council areas areas in Scotland. Besides, the longitudinal design with assessments at three time points, including an unexpectedly long-term follow-up at 60 months following baseline, will provide valuable opportunities to examine mechanistic changes across the sensitive teenage years as well as to disentangle causal relationships between hypothesised risk factors and longitudinal changes in mental health outcomes.

However, several limitations should be noted. Firstly, although recruitment efforts reached a diverse range of schools, the overall response rate and reasons for non-participation were not systematically recorded. It is therefore possible that self-selection bias may have influenced the characteristics of the final sample. Second, similar to other community-recruited samples, our sample was inevitably skewed towards the lower spectrums in terms of symptoms of depression and anxiety; future studies can consider using an enhanced recruitment approach to ensure that a larger proportion of adolescents with more severe symptoms are represented. 

Third, similar to many large-scale studies, our use of a comprehensive battery of measures – while offering a rich dataset – may have contributed to participant fatigue, potentially impacting the accuracy or completeness of responses. Although participants were allowed flexibility in completing online components, future iterations of the study may benefit from streamlining or rotating the measures to reduce burden.

Forth, there were notable levels of missing data in certain domains, including nearly 20% missing responses. While these proportions are not uncommon in adolescent self-report research, they do limit the interpretability of subgroup analyses. To reduce missing data in future research, strategies such as shorter, more focused data collection sessions and enhanced follow-up engagement should be considered to minimise participant fatigue and non-completion. Notably, in the analysis of follow-up data at subsequent time points, we have employed advanced statistical methods such as liner mixed-effects modelling to addressing missing data and longitudinal variability more comprehensively. 

Additionally, while some objective measures were used, in particular around biological and cognitive factors, the majority of the measures were self-report scales that might be susceptible to biases due to demand characteristics or social desirability effects. For instance, one core feature of depression is a tendency towards negative self-appraisal, which may influence how individuals perceive and report their own family’s mental health. As such, associations between family history and current symptoms should be interpreted cautiously, particularly given the cross-sectional nature of the baseline data and the lack of temporal clarity regarding directionality.

It is also worth noting that lower depression scores observed among adolescents over the age of 15 may be influenced by methodological factors such as school absence or early school leaving, which could disproportionately affect youth experiencing more severe mental health difficulties.

Lastly, upon reflection, the response options for demographic questions could be more comprehensive, for example, by including ‘transgender’ in the question regarding gender groups. Moreover, socioeconomic status (SES) data were not collected, which limited our ability to examine potential differences in symptoms by SES. Additionally, the sample was limited to Scottish young people, and future research targeting a wider geographical spread in the UK will help generalise the findings at a larger scale.

### 4.2. Conclusion

This report provides an overview of the EVA study, delving into its background, design, and methodology employed in the research. Additionally, the report elucidates the baseline demographic, clinical and mechanistic characteristics of participants and explores individual differences of the baseline measures of mental health, well-being and their potential underlying bio-psycho-social factors/ mechanisms based on key demographic and mental health characteristics, namely gender, age, and personal and familial history of mental health difficulties. Our key findings replicated previous findings in suggesting that females and those with a personal or familial history of mental health difficulties were particularly vulnerable with higher levels of depression and anxiety and lower levels of wellbeing. In terms of the hypothesised bio-psycho-social factors and mechanisms, these vulnerable groups were found to show poorer sleep quality, lower levels of resilience, and higher levels of rumination, stress, neuroticism, external shame, bullying experiences, neural-cognitive biases, and dysfunctional attitudes. It is also noteworthy that symptoms of depression and anxiety both increased with age and peaked around age 15; age was also associated with an increased risk for eating disorders. These findings highlight the need for preventative and early intervention approaches to consider individual differences and differences across sub-groups of populations.

## Ethics and consent

Ethical approval was obtained from the Research Ethics Committee at the University of Edinburgh (Reference no. STAFF115) on 10-05-2018 and the relevant local educational councils: Edinburgh (MG/AF, 17-05-2018), (Perth & Kinross Council, PD/CH, 26-09-2018), (Fife, SMcL/DCC/F17, 19-07-2018), Midlothian (23-03-2018). When the study was moved to the University of Reading, further ethics approval was obtained from the University of Reading (Reference no. UREC 23_22) on 19-09-2023.). Participants over the age of 16 years were asked to provide written informed consent for themselves; participants under the age of 16 years were asked to sign an assent form in addition to returning a parents’/guardians’ consent form.

## Data Availability

Open Science Framework: Emotional Vulnerability in Adolescents (EVA), https://doi.org/10.17605/OSF.IO/EKMAH ( Tariq, 2024) This project contains the following underlying data: EVA--Baseline Data. sav (Baseline data, Time 1 for EVA project) Open Science Framework: Emotional Vulnerability in Adolescents (EVA), https://doi.org/10.17605/OSF.IO/EKMAH ( Tariq, 2024) This project contains the following extended data: Final used from 01–19 PIS, consent, assent, demographic 12–15. pdf Final used from 01–19 PIS, consent, demographic 16+. pdf Online Survey Measures. pdf Data are available under the terms of the Creative Commons Attribution 4.0 International license (CC-BY 4.0).

## Software availability statement

Bristol Online Survey: https://www.onlinesurveys.ac.uk/

To ensure compliance with the guidelines, the following recommended software are Open-access alternatives that can be used in the absence of access to Online Surveys and perform equivalent functions:

1. **Google Forms:** Available at Google Forms
2. **Microsoft Forms:** Available at Microsoft Forms
