# Supplementary material for: Emotional Vulnerability in Adolescents (EVA) Longitudinal Study: Identifying individual differences in symptoms of adolescent depression and anxiety and their biopsychosocial mechanisms based on demographic and mental health characteristics
Source: Wellcome Open Res. 2025 Sep 1;9:510. Originally published 2024 Sep 4. [Version 2] doi: 10.12688/wellcomeopenres.22685.2 (PMC12640498; doi:10.12688/wellcomeopenres.22685.2)
Supplement: Peer review history

# Reviewer report: Reviewer response for version 2

**DOI:** 10.21956/wellcomeopenres.27370.r136770

**Published:** 2025-11-22

**Authors:** Unaiza Iqbal

**Affiliations:** 1 HELP University, Kuala Lumpur, Malaysia

I have thoroughly reviewed the revised version of this manuscript, and the authors have clearly and comprehensively addressed the previous reviewer comments/issues raised. The revisions made in this version have clearly enhanced the quality of the manuscript by improving clarity, coherence, and methodological transparency of the paper. Improvements made to the Introduction, Methods, Results, and Discussion sections have strengthened the overall scientific rigour and interpretative accuracy. The quality of the revisions presented in this version are satisfactory and I am happy to approve this version.

Is the work clearly and accurately presented and does it cite the current literature?

Yes

If applicable, is the statistical analysis and its interpretation appropriate?

Yes

Are all the source data underlying the results available to ensure full reproducibility?

Yes

Is the study design appropriate and is the work technically sound?

Yes

Are the conclusions drawn adequately supported by the results?

Yes

Are sufficient details of methods and analysis provided to allow replication by others?

Yes

Reviewer Expertise:

Clinical Psychology, human-animal interaction research.

I confirm that I have read this submission and believe that I have an appropriate level of expertise to confirm that it is of an acceptable scientific standard.

# Reviewer report: Reviewer response for version 2

**DOI:** 10.21956/wellcomeopenres.27370.r136773

**Published:** 2025-11-20

**Authors:** Stephanie Ameis

**Affiliations:** 1 University of Toronto Department of Psychiatry, Toronto, Ontario, Canada

The present report provides a well articulated background that clearly indicates the burden of emotional disorders in youth, need for early intervention and the evidence for multifactorial models of illness risk. The report aims to present the EVA protocol as well as present findings for the baseline sample. The goal of the study is stated as focused on identifying biopsychosocial variables linked to risk for poorer outcomes. Methods are clear in presenting the longitudinal study design, as well as pivots due to COVID. The current report presents data from the baseline sample of the EVA study (12-18 yo, recruited 2018-2019, across 12 schools in scotland and uk). The EVA study collected data at the 6 mo fu timepoint as well as a final follow up collection timepoint completed 5 years after baseline. The protocol mainly provides self reported measures, with the exception of actigraphy to track sleep and hair cortisol. Descriptive statistics in the current report present findings from a baseline sample of >400 high school students that participated in the study. The sample was reported as majority female and >70% white. Missingness ranged from ~20% on most variables to 40% (BMI). Findings indicate that depression and anxiety scores and well being scores reported in the sample are associated with age. Sleep indices of latency, efficiency, duration are also associated with age. Further, there were significant differences in self reported measures between males and females in the sample and for variables compared between those with versus without personal mental health, or family mental health history. The findings were interpreted as being consistent with previous literature and providing indication of the need for mental health intervention, particularly among females, and those with familial and personal mental health history indicative of ongoing risk for elevated scores. 

 While the current report is well written, the initial hypotheses of the EVA study and the hypotheses guiding the analyses of the present report are not provided. Further, the uniqueness of the current study and how this study addresses gaps and extends similar research is needed. That is, are there similar studies conducted in the local setting or globally that can be summarized in the introduction, to provide a sense of what the current evidence gaps are that the current study aims to address. 

 Given the level of missingness in the sample, it is important to indicate whether missingness was 'at random', and whether demographics of participants with missing scores are similar to the  overall sample. Further, what is the plan for the current and future studies using this sample wrt handling missingness. 

 The demographics of the sample include majority females and majority white. It would be important to understand how representative the current sample is of the schools they recruited from as well as the population of the surrounding areas in general. 

 some important factors are missing in a study focused on adolescent depression - pubertal markers. A rationale for this would be important. Further, given recruitment from schools, it would be important to justify why not leverage the opportunity to collect info from teachers on mental health of their students? Given females are over-represented, it may be that reporter bias contributes to elevated scores among females. further discussion of this is warranted - it is possible that there is something systematically different about females and males, such that females who have elevated distress may be more interested in participating in a mental health study, whereas males with elevated distress may be less likely, perhaps biasing the overall sample beyond differences in self report styles of females versus males alone. 

 Additional points:

 In the intro paragraph (1.1) it indicates that 75% of young people experiencing mental health difficulties by 24 - i think this statement would be more accurate and easier to interpret if sentence revised to: Among individuals who have experienced mental health difficulties, 75% experience onset by age 24. 

 section 1.5.2 it would be helpful to clarify whether stats represent global trends or are specific to developed counties -global north (ex 1/6 obese?)

 the present sample includes ~60% female and 70% white - how representative are these demographics of the schools where recruitment took place

 missingness ranged from 20 on a wide variety of items to 40% (BMI).  descriptive statistics for a number of the mechanistic variables appear to be missing in 1/4 of the sample

 3.2.2 and table 6 indicates positive corr bw age and sleep onset latency - i believe this would mean greater latency in older participants as opposed to the opposite which is indicated in paragraph 3.2.2

 in discussion, authors suggest that the current findings indicate need for targeted prevention and early intervention in schools and communities

Is the work clearly and accurately presented and does it cite the current literature?

Partly

If applicable, is the statistical analysis and its interpretation appropriate?

Partly

Are all the source data underlying the results available to ensure full reproducibility?

Yes

Is the study design appropriate and is the work technically sound?

Yes

Are the conclusions drawn adequately supported by the results?

Partly

Are sufficient details of methods and analysis provided to allow replication by others?

Yes

Reviewer Expertise:

child and youth psychiatry, neurodevelopment

I confirm that I have read this submission and believe that I have an appropriate level of expertise to confirm that it is of an acceptable scientific standard, however I have significant reservations, as outlined above.

# Reviewer report: Reviewer response for version 1

**DOI:** 10.21956/wellcomeopenres.24986.r113344

**Published:** 2024-12-23

**Authors:** Theresa Fleming

**Affiliations:** 1 School of Health, Victoria University of Wellington, Wellington, Wellington, New Zealand

This study explores a broad range of self-reported and observed factors and their associations with mental health symptoms in 425 adolescents in Scotland at baseline and at 6 and 60 months later. This paper reports the findings at baseline, describing the prevalence of  bio-psycho-social factors and symptoms of depression and anxiety and levels of well-being at baseline. It is a useful paper, however there is room to increase the importance of via editing.

 The article would be improved with editing to ensure support readability and precision. For example, there are minor grammatical errors, the use of past and future tense is mixed, several times the phrase 'on the contrary' is used where the point is not contrary to the prior one. In 1.3, should the wording be .. 'psychotherapeutic conditions'? In 2.1, do you mean 'formally' or 'formerly'? I assume '#8216; 2' and '#8216#true' in section 2.4.1 are not meant to be included? In your section 2.5, where you note that 'However, three participants were identified to have extremely high cortisol concentrations'.. do you mean extreme concentrations? one seems very low rather than high. 

 There is repetition and confusing detail in the introduction. For example, widely contrasting estimates of the prevalence of mental health problems are cited at several points in the opening paragraphs. The authors have noted BMI is a risk factor.  I assume this is high or low BMI - just make sure all these details are clear. They have identified those in the top and bottom 5% as problematic in one sentence and used absolute (rather than relative) definitions to indicate problematic BMI's elsewhere.

 There is a complex literature regarding associations between social media use and mental wellbeing. Are the authors confident they have summarized this in an accurate way?  

 A careful edit and check for precision throughout would be helpful.

**Methods:**

- A little more detail in the methods is needed. How were the areas, schools and participants selected? What was the response rate for schools and participants? How was personal and family mental health history assessed? Given the % reporting a 'family mental health history' and that findings are reported by this factor, it is important to explain what it is.
- Was social media use based on self report?
- How was ethnicity assessed? 
- Were the young people seen alone in the face to face meetings? 
- About how long did the assessments take?

I am not a biostatistician and can not provide detailed comment re statistical measures or tests used. 

**Results:**

- In the figures, make sure each label is clear.  I was a little confused by the label about 'depressive symptoms < the level of concern'. Could this be any clearer?
- When you are describing the results (e.g. 70.6% White), also report the % missing.
- It would be helpful to understand differences in symptoms by ethnicity and SES. 

In 3.1.3 you report that the adolescents where  relatively healthy with relatively low levels of depression and anxiety symptoms. However, over 20% indicate depressive symptoms and only 38% indicate 'normal' scores on anxiety. This does not seem relatively healthy. 

**Discussion:**

- In your consideration of strengths and weaknesses, Include the response rates (and reasons for non participation if possible) and any impact on the study. You have included a high number of measures.  Might this have influenced accuracy?
- There is a quite a lot of missing data - e.g. nearly 20% do not report an ethnicity and about 20% are missing for family and personal mental health history. This should be commented on. Do you have any plans to address this in future rounds?
- Is it possible to comment on findings by participant ethnicity or SES?
- Might lower rates of depression among teens over 15 reflect any methodological issues?  Eg less healthy teens leaving school earlier or being more likely to be absent on the day of the study?
-  A key feature of depression is feeling negatively about self, others, past, present and future.  Might this impact on report of self or family mental health history? I would expect this would be likely. This means statements such as 'those with personal or family mental illnesses are likely to be three to four times more susceptible to developing depression or anxious symptoms' need to be interpreted with caution, At baseline you do not know the direction of this relationship.

Is the work clearly and accurately presented and does it cite the current literature?

Partly

If applicable, is the statistical analysis and its interpretation appropriate?

I cannot comment. A qualified statistician is required.

Are all the source data underlying the results available to ensure full reproducibility?

Yes

Is the study design appropriate and is the work technically sound?

Yes

Are the conclusions drawn adequately supported by the results?

Yes

Are sufficient details of methods and analysis provided to allow replication by others?

Partly

Reviewer Expertise:

youth health and mental heatlh

I confirm that I have read this submission and believe that I have an appropriate level of expertise to confirm that it is of an acceptable scientific standard, however I have significant reservations, as outlined above.

# Response

**Published:** 2025-08-18

**Authors:** ASNEA TARIQ

**Affiliations:** School of Psychology & Clinical Language Sciences, University of Reading, Reading, England, UK

Thank you for taking the time to review our manuscript and for your helpful comments. We have resubmitted a revised manuscript, addressing your comments point by point below. Our responses are highlighted in blue for your convenience.  

**Reviewer Comment:** *“The article would be improved with editing to ensure support readability and precision. For example, there are minor grammatical errors, the use of past and future tense is mixed, several times the phrase 'on the contrary' is used where the point is not contrary to the prior one… There is repetition and confusing detail in the introduction. For example, widely contrasting estimates of the prevalence of mental health problems are cited at several points in the opening paragraphs. The authors have noted BMI is a risk factor. I assume this is high or low BMI - just make sure all these details are clear… There is a complex literature regarding associations between social media use and mental wellbeing. Are the authors confident they have summarized this in an accurate way?”*  

**Response:**

 Thank you for your constructive feedback. In response, we have made substantial revisions throughout the Introduction to improve clarity, readability, and precision. These changes include:

- We have carefully reviewed and corrected grammatical errors, punctuation, and inconsistencies in tense use across the sections to enhance precision.
- We have replaced instances of “on the contrary” with more appropriate connectors to ensure alignment with the logical flow of the argument.
- We have streamlined the opening sections (1.1 and 1.2) to avoid repetitive or overly contrasting prevalence estimates and have now provided more contextualised statistics to support the rationale without confusion.
- We have now specified that both high (overweight/obese) and low (underweight) BMI are considered risk factors and defined BMI percentiles (top or bottom 5%) using appropriate references.
- We have revised the subsection on social media use (Section 1.5.2) to more accurately reflect the current evidence base and acknowledged the complexity and limitations of the literature. We have exercised caution in interpreting causal claims and ensured that gender-specific findings were appropriately contextualised.
- Throughout the introduction (Section 1), we have refined sentence structure and vocabulary for improved readability without altering the substantive content or structure. Redundancies have been eliminated, and clearer transitions have been added between paragraphs to enhance readability.

 
**Reviewer Comment:** *“A little more detail in the methods is needed. How were the areas, schools and participants selected? What was the response rate for schools and participants? How was personal and family mental health history assessed? Given the % reporting a 'family mental health history' and that findings are reported by this factor, it is important to explain what it is. Was social media use based on self-report? How was ethnicity assessed? Were the young people seen alone in the face-to-face meetings? About how long did the assessments take?”*  

**Response: ** We have now clarified these details in the Methods section. Specifically, we have added details in Sections 2.2, 2.3, and 2.4.6 regarding the study procedure, assessment duration, and the assessment of mental health history, ethnicity, and social media use.  

**Reviewer Comment: ** “ *• In the figures, make sure each label is clear. I was a little confused by the label about 'depressive symptoms < the level of concern'. Could this be any clearer?* *• When you are describing the results (e.g., 70.6% White), also report the % missing.*

*• It would be helpful to understand differences in symptoms by ethnicity and SES.*

*• In 3.1.3 you report that the adolescents were relatively healthy with relatively low levels of depression and anxiety symptoms. However, over 20% indicate depressive symptoms, and only 38% indicate 'normal' scores on anxiety. This does not seem relatively healthy.”* **Response: ** Thank you for these helpful suggestions. We have addressed each point as follows:

- We agree that the label "depressive symptoms < the level of concern" was ambiguous. As we were unable to edit the embedded text within the image directly, we have updated the figure title and caption to clearly reflect that scores above a specified threshold indicate clinical concern for depressive symptoms (Mood and Feelings Questionnaire; SMFQ; Angold et al., 1995). Similar clarifications have also been applied to the anxiety (Generalised Anxiety Disorder Screener; Spitzer et al., 2006) and well-being (Short Warwick-Edinburgh Mental Well-being Scale; SWEMWBS) figures.
- The percentage of missing data is already included in the demographic table (Table 2), which reports the proportion of participants for whom each variable was not available.
- We appreciate the suggestion to examine differences in symptoms by ethnicity and socioeconomic status (SES). Unfortunately, SES information was not collected in the present study, which we now acknowledge as a limitation in the revised manuscript. However, in line with the reviewer’s recommendation, we conducted additional analyses to examine ethnic group differences (Section 3.2.6). Independent samples t-tests were conducted to compare White and non-White participants across all key study variables. These analyses revealed no statistically significant differences between groups, and the results are now summarized in Table 10.
- We appreciate the reviewer highlighting the inconsistency in describing the sample as “relatively healthy.” In response, we have revised the text in Section 3.1.3 to provide a more accurate reflection of the data. The updated interpretation acknowledges that while many participants scored below clinical thresholds, a substantial proportion reported elevated symptoms, particularly for anxiety and depression, in line with population-level community samples.

 
**Reviewer Comment:** “ *• In your consideration of strengths and weaknesses, include the response rates (and reasons for non-participation if possible) and any impact on the study. You have included a high number of measures.* *Might this have influenced accuracy?* *• There is a quite a lot of missing data – e.g. nearly 20% do not report an ethnicity and about 20% are missing for family and personal mental health history. This should be commented on. Do you have any plans to address this in future rounds?* *• Is it possible to comment on findings by participant ethnicity or SES?* *• Might lower rates of depression among teens over 15 reflect any methodological issues? E.g., less healthy teens leaving school earlier or being more likely to be absent on the day of the study?* *• A key feature of depression is feeling negatively about self, others, past, present and future. Might this impact on report of self or family mental health history? I would expect this would be likely. This means statements such as 'those with personal or family mental illnesses are likely to be three to four times more susceptible to developing depression or anxious symptoms' need to be interpreted with caution. At baseline you do not know the direction of this relationship”.*  

**Response: ** We thank the reviewer for these thoughtful and constructive comments, which have led to several important additions and clarifications in **Section 4.1 (Strengths and Limitations)** of the revised manuscript. We have now reflected on the potential impact of administering a large number of measures and acknowledged that participant burden may have influenced response accuracy. The levels of missing data have now been explicitly addressed.   We appreciate the reviewer’s suggestion regarding the observed lower depressive symptom scores among older adolescents. We now acknowledge that this trend may be influenced by methodological factors, such as selective school dropout or absence among more vulnerable students. This possibility has been incorporated into Section 4.1 as a potential limitation of the current cross-sectional baseline findings. Finally, we agree that depressive cognitive distortions may affect how individuals report their own or their family members’ mental health histories. Accordingly, we have revised the relevant interpretations to reflect this potential reporting bias and to emphasise the exploratory and non-causal nature of these baseline associations. As above, we have now added results around differences by ethnicity in the Results section.
